# A Peroxygenase‐Alcohol Dehydrogenase Cascade Reaction to Transform Ethylbenzene Derivatives into Enantioenriched Phenylethanols

**DOI:** 10.1002/cbic.202200017

**Published:** 2022-01-27

**Authors:** Xiaomin Xu, Hugo Brasselet, Ewald P. J. Jongkind, Miguel Alcalde, Caroline E. Paul, Frank Hollmann

**Affiliations:** ^1^ Department of Biotechnology Delft University of Technology 2629HZ Delft (The Netherlands; ^2^ Department of Biocatalysis Institute of Catalysis, CSIC 28049 Madrid Spain

**Keywords:** alcohol dehydrogenases, asymmetric reduction, bienzymatic cascades, oxyfunctionalisation, peroxygenases

## Abstract

In this study, we developed a new bienzymatic reaction to produce enantioenriched phenylethanols. In a first step, the recombinant, unspecific peroxygenase from *Agrocybe aegerita* (r*Aae*UPO) was used to oxidise ethylbenzene and its derivatives to the corresponding ketones (prochiral intermediates) followed by enantioselective reduction into the desired (*R*)‐ or (*S*)‐phenylethanols using the (*R*)‐selective alcohol dehydrogenase (ADH) from *Lactobacillus kefir* (*Lk*ADH) or the (*S*)‐selective ADH from *Rhodococcus ruber* (ADH‐A). In a one‐pot two‐step cascade, 11 ethylbenzene derivatives were converted into the corresponding chiral alcohols at acceptable yields and often excellent enantioselectivity.

## Graphical abstract

**A bienzymatic cascade** to access complementary chiral phenylethanols from simple ethylbenzene derivatives is presented, combining peroxygenase‐catalysed oxyfunctionalisation to the corresponding acetophenone with alcohol dehydrogenase‐catalysed enantioselective reduction.

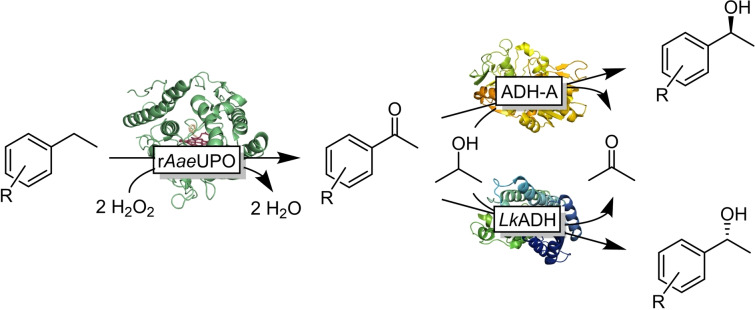

## Introduction

**Scheme 1:**
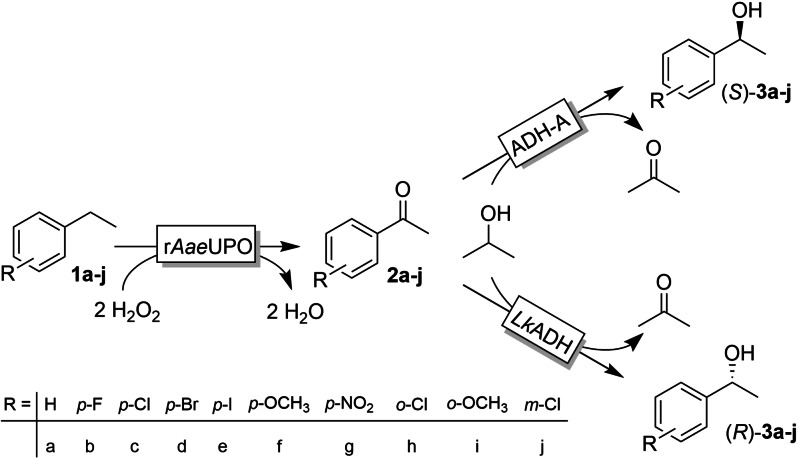
Envisioned bienzymatic cascade to access both enantiomers of phenylethanols from non‐functionalised ethylbenzenes. The first step comprises a peroxygenase from *Agrocybe aegerita*, r*Aae*UPO‐catalysed double hydroxylation to the prochiral acetophenones followed by stereoselective reduction to (*R*)‐phenylethanol (catalysed by the alcohol dehydrogenase from *Lactobacillus kefir*, *Lk*ADH) or to the (*S*)‐alcohol (catalysed by the alcohol dehydrogenase from *Rhodococcus ruber*, ADH‐A).

Oxyfunctionalisation reactions (i. e. the insertion of O‐atoms into C−H−, C=C‐bonds) represent a central theme in organic synthesis and particularly in the manufacture of pharmaceutically active ingredients^[1]^ and the quest for efficient (economically viable and environmentally acceptable) syntheses continues. The synthesis of chiral 1‐phenylethanol derivatives for example has been the focus of various research groups. Chiral, α‐functionalised benzene derivatives are found in approximately 15 % of the 2020 top selling small molecule drugs^[2]^ and various synthetic routes such as the enantioselective reduction of prochiral acetophenone derivatives^[3]^ or the (dynamic) kinetic resolution of racemic 1‐phenylethanols^[4]^ have been established. Envisioning ethylbenzene derivatives as starting materials (i. e. obtaining chiral 1‐phenylethanols through selective benzylic hydroxylation), heme‐dependent enzymes appear to be the catalysts of choice.^[5]^ One shortcoming of current enzymatic enantioselective hydroxylation, however, is a lack of enantiocomplementary oxygenases, often limiting access to only one enantiomer. Inspired by recent contributions from the groups of Flitsch^[6]^ and Kroutil^[7]^ we envisioned a bienzymatic cascade to give access to both enantiomers of 1‐phenylethanol and its derivatives. Particularly, we propose to combine peroxygenase‐catalysed oxyfunctionalisation yielding prochiral ketones followed by enantioselective alcohol dehydrogenase‐catalysed reduction to the corresponding alcohols. In particular, we used the recombinant, evolved peroxygenase from *Agrocybe aegerita* (r*Aae*UPO)^[8]^ for the benzylic oxidation via (*R*)‐1‐phenylethanol (derivatives) to the acetophenones of interest. To catalyse the stereoselective ketoreduction reaction we used either the (*R*)‐selective alcohol dehydrogenase from *Lactobacillus kefir* (*Lk*ADH)^[9]^ or the (*S*)‐selective alcohol dehydrogenase from *Rhodococcus ruber* (ADH‐A) (Scheme 1).^[10]^

## Results and Discussion

In a first set of experiments we established the ‘thorough oxidation’ of various ethylbenzene derivatives to the corresponding acetophenone derivatives (Table 1). Expectedly, the first oxidation step proceeded highly enantioselectively yielding the (*R*)‐phenylethanol derivative (Table S1). Generally, the starting materials tested were smoothly converted into the corresponding ketone products with the exception of *p*‐iodo‐ **1 e** and *p*‐methoxyethylbenzene **1 f** (Table 1). In case of sluggish oxidation rates, increasing the r*Aae*UPO‐concentration resulted in complete oxidation e. g. of *p*‐iodoethylbenzene **1 e** to the corresponding ketone product. Unfortunately, the mass balance was generally not closed, which can be attributed to either the experimental setup allowing for evaporation of the volatile reagents or the poor solubility of several reagents that led to quantification issue (see Supporting Information).

**Table 1:** Selection of ethylbenzene derivatives converted by r*Aae*UPO.

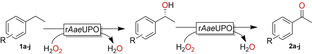			
Starting material	Starting material **1** [mM]	Alcohol [mM]	Ketone **2** [mM]
**1 a**	0	0	35.8±1.5
**1 b**	0	0	41±4.2
**1 c**	0	0	46.4±2.6
**1 d**	0.4±0.2	0.4±0.4	42.3±32.7
**1 e**	20.3±17.5	0.7±0.1	18.5±10.7
**1 e** ^[a]^	0	0	46.6±4.5
**1 f**	0	30.7±4.0	11.5±1.5
**1 g**	0.5±0.5	0	21.8±1.9
**1 h**	0.7±0.7	2.2±1.3	34.9±3.1
**1 i**	7.6±3.8	7±0.3	29.8±0.9
**1 j**	1.1±0.2	2.1±0.5	33.6±1.1

Reaction conditions: [substrate **1**]=50 mM, [r*Aae*UPO]=2 μM, buffer: 50 mM KPi pH 7 containing 10 % v/v acetonitrile, 25 °C, H_2_O_2_ dosing rate: 20 mM h^−1^, reaction time: 6 h. Experiments were performed as duplicates. [a] [r*Aae*UPO]=6 μM.

It should be mentioned that the reaction conditions chosen in these experiments, particularly the enzyme concentration and H_2_O_2_ feeding strategy have not been optimised neither for space‐time‐yields nor for enzyme utilisation. However, already under these non‐optimised conditions turnover numbers for the peroxygenase up to 42300 (corresponding to approx. 100 g_prod_×g_*Aae*UPO_^−1^) have been achieved.

**Figure 1:**
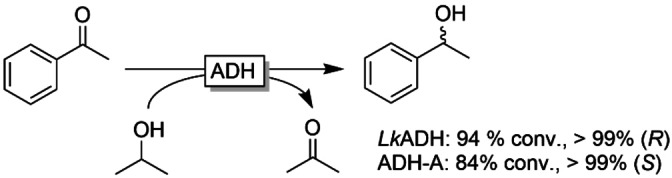
Stereoselective reduction of acetophenone using two enantiocomplementary ADHs. Conditions: [acetophenone]=15 mM, [NAD(P)H]=0.1 mM, 10 % v/v 2‐propanol, [MgCl_2_]=2 mM, *Lk*ADH/ADH‐A: 50 μL cell free extract or 20 mg lyophilised cells, 50 mM KPi buffer pH 7, 30 °C, 600 rpm, 24 h.

Next, we validated the possibility of enantioselective reduction of acetophenone with *Lk*ADH and ADH‐A (Figure 1). For reasons of simplicity, we chose for a substrate‐coupled NAD(P)H regeneration approach using isopropanol as sacrificial reductant.

Having established the individual components of the envisioned cascade, we further combined both enzymatic steps in one pot. In a first try, we tested both enzymatic steps concurrently (one‐pot one‐step) using ethyl benzene as starting material. At first sight, the cascade was successful producing significant amounts of the desired phenylethanol (Table 2). However, a closer inspection of the optical purity of the alcohol products revealed a poor ee‐value of the (ADH‐A‐derived) (*S*)‐phenylethanol **3 a** of 19 % ee. We observed a significant amount of r*Aae*UPO‐derived (*R*)‐phenylethanol had not been thoroughly oxidised to the ketone, thereby contaminating the ADH‐A‐derived (*S*)‐enantiomer product. A plausible explanation for the decreased r*Aae*UPO‐activity is the presence of isopropanol in the reaction, which competes with phenylethanol for r*Aae*UPO‐catalysed oxidation thereby slowing down the oxidation steps of the cascade. Furthermore, the accumulating acetone decreases the thermodynamic driving force for the ADH‐catalysed reduction step. Apparently, the oxidative and reductive partial steps are not compatible for a one‐pot one‐step procedure.

**Table 2:** Results from the one‐pot one‐step system.^[a]^

ADHs	Phenylethanol **3 a** [mM]	ee [%]
*Lk*ADH	11.3±0.1	98 (*R*)
ADH‐A	15.1±1.2	19 (*S*)

[a] Reaction conditions: [ethylbenzene **1 a**]=50 mM, [NAD(P)H]=0.1 mM, 10 % v/v 2‐proponol, [MgCl_2_]=2 mM, [r*Aae*UPO]=2 μM, *Lk*ADH or ADH‐A=50 μL cell‐free extract, H_2_O_2_ dosing rate: 10 mM h^−1^ (10 h), 50 mM KPi buffer pH 7, 30 °C, 600 rpm. Data presented are averages of triplicate experiments.

**Figure 2:**
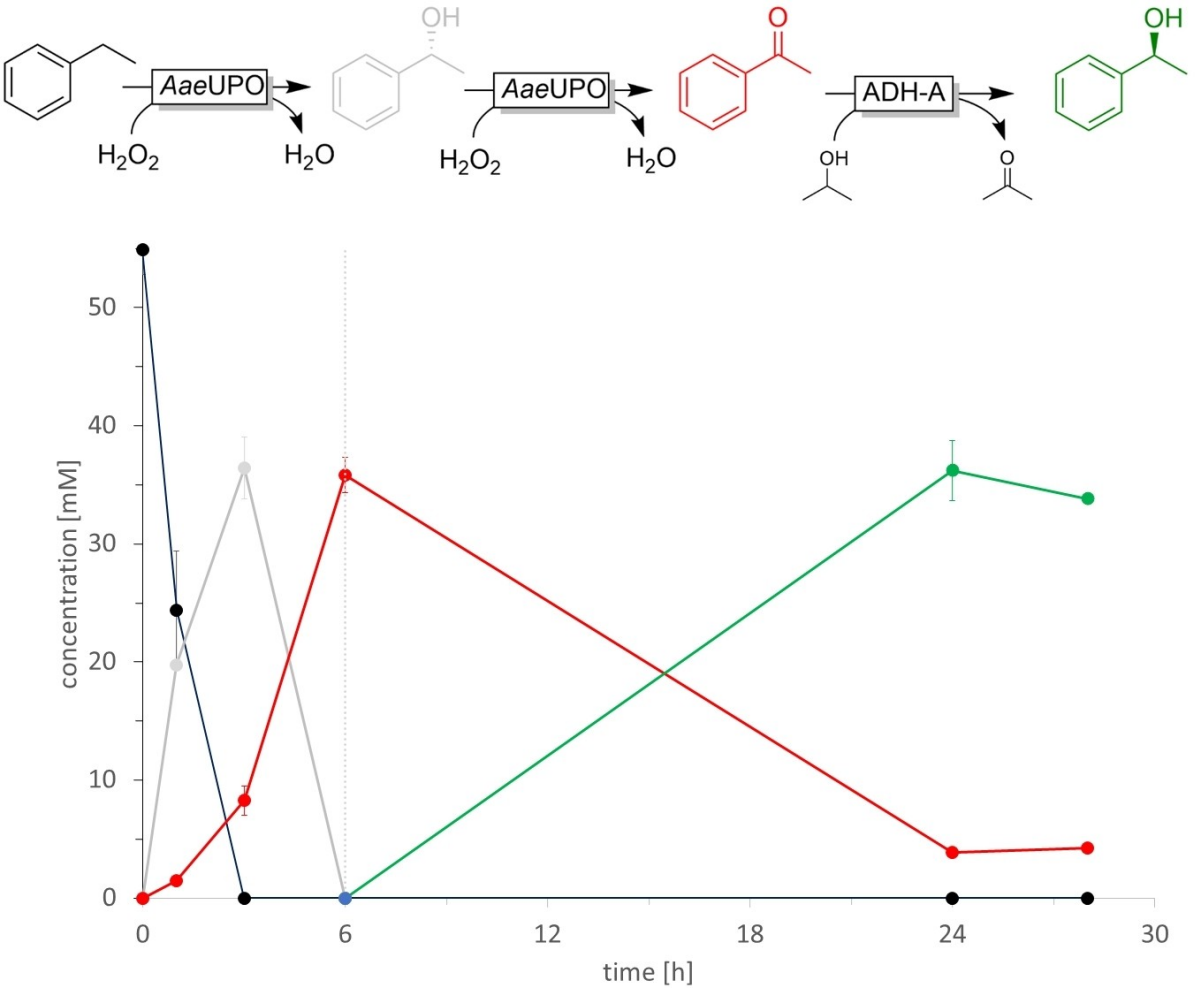
Representative time course of the bienzymatic one‐pot two‐step cascade converting ethylbenzene **1 a** to (*S*)‐phenylethanol **3 a**. Reaction conditions: [ethylbenzene **1 a**]=50 mM, [r*Aae*UPO]=2 μM, buffer: 50 mM KPi pH 7 containing 10 % (v/v) of acetonitrile, 25 °C. The reaction was initiated by addition of H_2_O_2_ (20 mM h^−1^), after 6 h H_2_O_2_ addition was stopped and the reaction mixture was supplemented with [NAD(P)H]=0.1 mM, [MgCl_2_]=2 mM, 10 % v/v 2‐proponol, ADH‐A (100 μL cell free extract) and incubated overnight.. Experiments were performed as triplicates.

We therefore drew our attention to a one‐pot two‐step procedure, in which first the r*Aae*UPO‐catalysed conversion of ethylbenzenes to the corresponding acetophenone derivatives occurs, followed by the ADH‐catalysed reduction to the chiral alcohols. Concretely, in a first phase the r*Aae*UPO‐catalysed ethylbenzene (50 mM) oxyfunctionalisation was conducted for 6 h with a H_2_O_2_‐addition rate of 20 mM h^−1^. This was followed by the addition of the ADH catalysts together with isopropanol (as sacrificial reductant) and incubation overnight. A first experiment combining r*Aae*UPO with ADH‐A (Figure 2) gave promising results converting initial 55 mM of ethyl benzene into 1‐phenylethanol (35.8 mM final concentration).

As this strategy proved to be successful, we further applied it for the conversion of the starting materials evaluated previous (*vide supra*). As shown in Table 3, the majority of ethylbenzene starting materials **1** was successfully converted into the corresponding (*R*)‐ or (*S*)‐phenylethanols **3** at reasonable yields and generally good optical purities (Table 3). One notable exception was the *o*‐methoxy derivate **1 i** where in both cases low alcohol concentrations of the (*R*)‐alcohol were observed. Although a better understanding of the issue with this substrate necessitates further experiments, it may be assumed that the *Lk*ADH and ADH‐A exhibited low to no activity towards the ketone intermediate **2 i**.^[11]^ We currently cannot explain the (*R*) enantiomeric reduction product for the *p*‐methoxy derivative **3 f** with ADH‐A.

**Table 3:** Results from the one‐pot two‐step conversion of ethylbenzene derivatives **1 a**–**j** to the corresponding phenylethanols **3 a**–**j**.

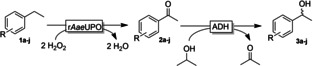				
Starting	ADH‐A		*Lk*ADH	
material	Alcohol **3** [mM]	ee [%]	Alcohol **3** [mM]	ee [%]
**1 a**	36.2±2.6	>99 (*S*)	40.1±1.0	>99 (*R*)
**1 b**	38.6±3.2	>99 (*S*)	36.7±3.3	>99 (*R*)
**1 c**	23.9±11.5	98 (*S*)	34.0±8.5	>99 (*R*)
**1 d**	36.7±0.6	>99 (*S*)	38.2±0.8	>99 (*R*)
**1 e** ^[a]^	33.5±6.7	>99 (*S*)	30.9±1.2	>99 (*R*)
**1 f**	37.9±0.8	>99 (*R*)	37.6±1.17	>99 (*R*)
**1 g**	24.6±4.8	>99 (*S*)	13.1±1.9	>99 (*R*)
**1 h**	27±1.3	>99 (*S*)	35.8±5.4	91(*R*)
**1 i**	5.2±0.1	>99 (*R*)	5.4±0.3	>99 (*R*)
**1 j**	47.7±4.9	92 (*S*)	51.7±0.9	>99 (*R*)

Reaction conditions: [substrate **1**]=50 mM, [r*Aae*UPO]=2 μM, buffer: 50 mM KPi pH 7 containing 10 % v/v of acetonitrile, 25 °C, H_2_O_2_ dosing rate: 20 mM h^−1^, reaction time: 6 h. For the second step the reaction mixture was complemented with [NAD(P)H]=0.1 mM, [MgCl_2_]=2 mM, 10 % v/v 2‐propanol, *Lk*ADH or ADH‐A (100 μL cell‐free extract), and incubated overnight. Experiments were performed as triplicates. [a]: [r*Aae*UPO]=6 μM.

**Figure 3:**
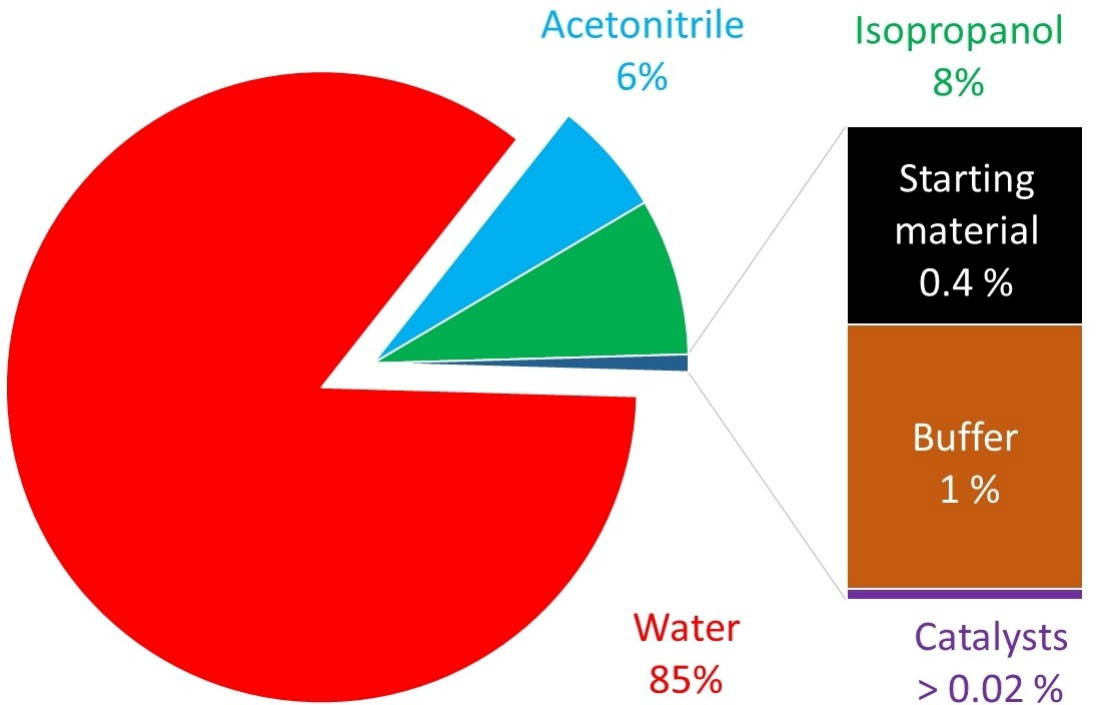
E‐Factor analysis of the bienzymatic conversion of *p*‐chloroethylbenzene **1 c** to (*S*)‐*p*‐chlorophenylethanol **3 c**.

Overall, we have established a bienzymatic cascade reaction to obtain both enantiomers of a range of phenylethanol derivatives. To assess the environmental impact of the reaction and to identify the bottlenecks of the current reaction system we performed an E‐factor analysis^[12]^ of the reaction (at the example of *p*‐chloroethylbenzene **1 c**, Figure 3).

The wastes generated in the current setup correspond to approx. 262 kg_waste_×kg^−1^_product_, of which 99 % are caused by the (co‐)solvents. Non reacted starting material and ketone intermediate contribute to less than 0.5 % (E‐factor contribution of 0.37 kg×kg^−1^), and the contribution of the catalysts (r*Aae*UPO, ADH‐A, NAD: approx. 0.02 kg×kg^−1^) or of the buffer salts are negligible. Evidently a simple E‐factor analysis does not give a complete picture as it neglects the pre‐history of the reagents and catalysts used as well as energy requirements^[13]^ and therefore can only give a first indication about the real environmental impact. Nevertheless, the dominance of (co‐)solvents points out in which direction further improvements are necessary. First, the product concentration needs to be increased dramatically.^[14]^ Fed‐batch strategies adding the reagents over time appear particularly suitable to obtain high concentrations. Possibly, this will require higher concentrations of the cosolvent (acetonitrile or environmentally more acceptable alternatives)^[15]^ to reduce the water consumption. In this way, the reaction system will also meet the requirements formulated by Huisman and co‐workers for the biocatalytic synthesis of APIs.^[16]^ Isopropanol also contributed significantly to the E‐factor (21 kg×kg^−1^ corresponding to 8 % of the overall E‐factor). Considering that the isopropanol's main function was to shift the equilibrium of the ADH‐catalysed reduction reaction, smarter, irreversible regeneration systems for the reduced nicotinamide cofactor^[17]^ or switching to enzyme‐coupled regeneration approaches^[18]^ appear attractive to reduce this contribution.

## Conclusion

Overall, we have established a biocatalytic access to complementary chiral phenylethanols from simple ethylbenzene derivatives by combining the peroxygenase‐catalysed thorough oxidation of alkyl benzenes to the corresponding ketones, followed by stereoselective alcohol dehydrogenase‐catalysed synthesis of enantiomerically enriched alcohols. Further steps will comprise broadening the substrate scope (e. g. to heteroaromatic starting materials) and increasing the substrate loading in order to turn this approach into an economically and ecologically interesting methodology for the synthesis of fine chemicals and APIs.

## Experimental Section

**Enzyme preparation**: Recombinant expression and purification of the evolved unspecific peroxygenase mutant from *A. aegerita* in *P. pastoris* was performed following a previously described procedure.^[11]^

**Acetophenone derivatives production**: In a 20 mL glass vial the reaction mixture (6.5 mL total volume) contained 2 μM r*Aae*UPO, 50 mM substrate, and 10 % v/v CH_3_CN in 50 mM KPi buffer pH 7.0. The reaction started by addition of H_2_O_2_, which was supplied with a continuous flow rate of 20 mM/h and run at room temperature (about 20 °C), 600 rpm, 6 h.

**Acetophenone reduction**: In a 1.5 mL GC glass vial the reaction mixture (700 μL total volume) contained 2 mM MgCl_2_, 0.1 mM NAD(P)H,50 μL cell‐free extract or 20 mg lyophilised cells of *Lk*ADH or ADH‐A, 10 % v/v 2‐propanol and 15 mM acetophenone in 50 mM KPi buffer pH 7.0. The reaction was run at 30 °C, 600 rpm, overnight.

**One‐pot one‐step system**: In a 1.5 mL GC glass vial the reaction mixture (700 μL total volume) contained 2 μM r*Aae*UPO, 50 mM ethylbenzene, 2 mM MgCl_2_, 0.1 mM NAD(P)H, 10 % v/v 2‐propanol, 50 μL of ADH‐A or *Lk*ADH cell‐free extract in 50 mM KPi buffer pH 7.0. The reaction was started by addition of H_2_O_2_, which was supplied with a continuous flow rate 10 mM/h with 10 h, and run at 30 °C, 600 rpm, 24 h.

**One‐pot two‐step system**: In a 20 mL glass vial the reaction mixture (6.5 mL total volume) contained 2 μM r*Aae*UPO, 50 mM substrates, 10 % v/v CH_3_CN in 50 mM KPi buffer pH 7.0. The reaction was started by addition of H_2_O_2_, which was supplied with a continuous flow rate of 20 mM/h and run at room temperature (about 20 °C), 600 rpm. After 6 h, 365 μL of the reaction mixture was taken into a 1.5 mL glass vial, then 2 mM MgCl_2_, 0.1 mM NAD(P)H, 10 % v/v 2‐propanol, 100 μL of ADH‐A or *Lk*ADH cell‐free extract was added and run at 30 °C, 600 rpm, overnight.

Thermomixers were used for controlling temperature and shaking. For each sampling over time, 100 μL of reaction mixture was extracted with 500 μL ethyl acetate containing 5 mM *n*‐octanol as internal standard. The extraction was then dried by MgSO_4_ and analysed by achiral and chiral gas chromatography. Details of gas chromatography and temperature profiles are shown in the Supporting Information.

## Conflict of interest

The authors declare no conflict of interest.

1

## Supporting information

As a service to our authors and readers, this journal provides supporting information supplied by the authors. Such materials are peer reviewed and may be re‐organized for online delivery, but are not copy‐edited or typeset. Technical support issues arising from supporting information (other than missing files) should be addressed to the authors.

Supporting Information

## Data Availability

The data that support the findings of this study are available in the supplementary material of this article.
