# Supplementary material for: A Peroxygenase‐Alcohol Dehydrogenase Cascade Reaction to Transform Ethylbenzene Derivatives into Enantioenriched Phenylethanols
Source: Chembiochem. 2022 Jan 27;23(6):e202200017. doi: 10.1002/cbic.202200017 (PMC9306878; doi:10.1002/cbic.202200017)
Supplement: Supplementary file 1 — Supporting Information [file CBIC-23-0-s001.pdf]

ChemBioChem

Supporting Information

A Peroxygenase-Alcohol Dehydrogenase Cascade Reaction to Transform Ethylbenzene Derivatives into Enantioenriched Phenylethanols

Xiaomin Xu, Hugo Brasselet, Ewald P. J. Jongkind, Miguel Alcalde, Caroline E. Paul, and Frank Hollmann*

Contents

1. General information **2**
2. Enzymes **2**
3. Enzymatic reaction conditions **2**
3.1. Synthesis of acetophenone derivatives 2
3.2. Acetophenone reduction by ADHs 3
3.3. One-pot one-step system 3
3.4. One-pot-two-steps system 3
4. Analytical procedures **3**
4.1. Determination of r*Aae*UPO activity 3
4.2. SDS-PAGE 3
4.3. GC measurements 4
5. GC chromatograms **7**
6. GC-MS chromatograms **27**
7. References **27**

1. General information

Unless otherwise mentioned, all chemicals were purchased from Sigma-Aldrich, TCI-Europe or abcr GmbH, and used without further purification. Columns and column material for enzyme purification were purchased from GE Healthcare

2. Enzymes

The PaDaI variant of the unspecific peroxygenase (r*Aae*UPO) from *Agrocybe aegerita* was expressed in *Pichia pastoris* as previously described.[1]

The alcohol dehydrogenases ADH-A from *Rhodococcus ruber* and *Lk*ADH from *Lactobacillus brevis*, were recombinantly produced in *E.coli* (Figure S1) as previously described.[2]

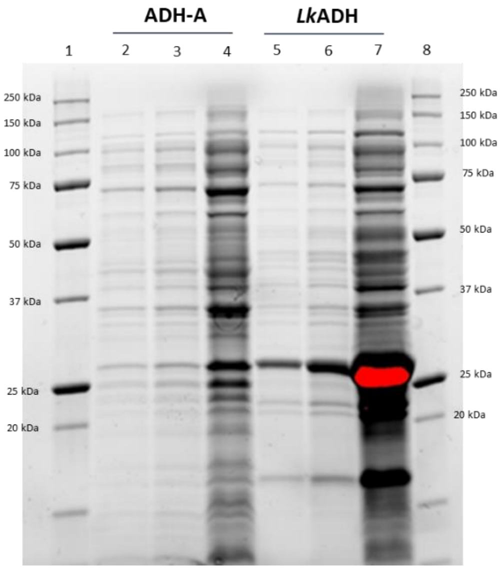

Figure S1. SDS-PAGE of ADH-A and *Lk*ADH cell free extracts used. Line 1 and 8: Marker; lines 2-4: ADH-A at 3 dilutions (20×, 10×, 1×); lines 5-7: *Lk*ADH at three dilutions (20×, 10×, 1×).

3. Enzymatic reaction conditions

3.1. Synthesis of acetophenone derivatives

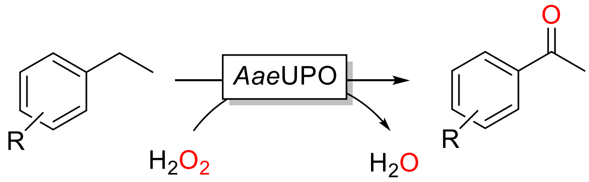

Scheme S1. Synthesis of acetophenone derivatives.

The reactions were performed in 20 mL glass vials at room temperature, under ambient atmosphere. The ethylbenzene derivatives (0.325 mmol) were diluted in CH_3CN (650 µL), and mixed with 4850 µL 50 mM KPi buffer, pH 7.0 and 2 µM r*Aae*UPO (83.88 µM stock, 160 µL). H_2O_2 (1 M in MilliQ) was added in the vial *via* a tube connected to a syringe pump (20 mM/h, 130 µL/h). The system was closed to avoid evaporation as much as possible. A ThermoMixer C from Eppendorf (Figure S2) was used for the reactions at a speed of 600 rpm.

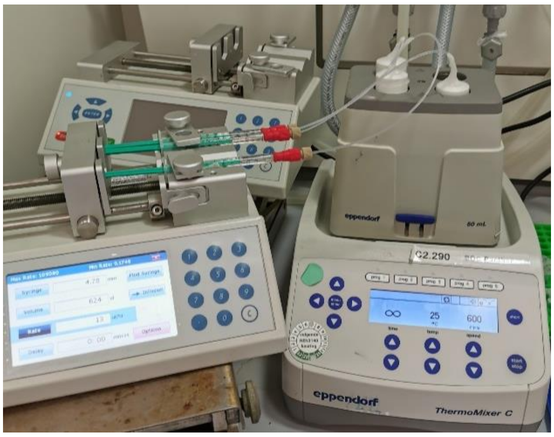

Figure S2. Reaction set-up with the syringe pump and ThermoMixer C.

3.2. Acetophenone reduction by ADHs

The reactions were performed in GC glass vials of 1.5 mL (Figure S2). Containing 2 mM $MgCl_2$, 0.1 mM NAD(P)H, ADH-A/*Lk*ADH: 50 µL cell free extract/20 mg dry cell, 10% v/v 2-propanol and 15 mM acetophenone (total volume: 700 µL). A ThermoMixer C from Eppendorf was used to control the temperature at 30 °C and speed at 600 rpm.

3.3. One-pot one-step system

The reactions were performed in a 1.5 GC glass vials a solution (700 µL total volume) containing 2 µM r*Aae*UPO, 50 mM ethylbenzene, 2 mM $MgCl_2$, 0.1 mM NAD(P)H, 10% v/v 2-propanol, 50 µL of ADH-A/*Lk*ADH cell free extract in 50 mM KPi buffer (pH 7.0). H_2O_2 (1 M in MilliQ) was added in the vial *via* a tube connected to a syringe pump (10 mM/h, 7 µL/h, 10 h). The reaction was running 24 h. A ThermoMixer C from Eppendorf was used to control the temperature at 30 °C and speed at 600 rpm.

3.4. One-pot-two-steps system

The reactions were performed in 20 mL glass vials starting at room temperature. The ethylbenzene derivatives (0.325 mmol) were diluted in CH_3CN (650 µL), and mixed with 4850 µL 50 mM KPi buffer, pH 7.0 and 2 µM r*Aae*UPO (83.88 µM stock, 160 µL). H_2O_2 (1 M in MilliQ) was added in the vial *via* a tube connected to a syringe pump (130 µL/h). After 6 h, 365 µL of the reaction mixture was taken into a 1.5 mL glass vial, 2 mM $MgCl_2$, 0.1 mM NAD(P)H, 10% v/v 2-propanol, 100 µL of ADH-A/*Lk*ADH cell free extract was added. The reaction was running overnight. A ThermoMixer C from Eppendorf was used to control the temperature at 30 °C and speed at 600 rpm.

4. Analytical procedures

4.1. Determination of r*Aae*UPO activity

The volumetric activity was determined *via* ABTS-assay at 25 °C. ABTS-assay was performed in sodium citrate buffer (50 mM, pH 4.4) containing H_2O_2 (2.0 mM), ABTS (0.5 mM) and a diluted enzyme solution. The oxidation of ABTS was monitored by absorption change at 420 nm (ε_{420} = 36.0 mM^{-1} cm^{-1}). Reactions were performed in triplicates.

4.2. SDS-PAGE

10 µL Protein samples were diluted with distilled water and mixed with 9.5 µL staining agent (Laemmli Sample buffer 4x) and 0.5 µL reducing agent (DDT 20x) to a final volume of 20 µL. After heat incubation the samples, 10 µL samples were loaded onto the gel. A precast gel was used (Criterion^{TM} TGX Stain-Free) with 1x TGS running buffer. 10 µL of marker solution (Precision Plus ProteinTM Unstained

Standard) was applied for every run. As running conditions, a constant voltage of 200 V and a starting current of 165 mA per gel with a running time of 45 min were applied.

4.3. GC measurements

GC measurements were performed on a Shimadzu GC-14A/FID or Shimadzu GC-2010 plus/FID equipped with different columns (Table S1 and S2). The reactions were stopped at different time points by addition of ethyl acetate containing n-octanol (5 mM) as internal standard. After extraction and centrifugation, the organic phase was dried with magnesium sulfate and analysed *via* gas chromatography. All concentrations reported, are based on calibration curves obtained from authentic and synthesised standards.

Table S1. **.** GC analytical data with achiral columns

Compound	GC column	Oven temperature program	Retention time
Ethylbenzene<br>1-Phenylethanol<br>Acetophenone	CP-Wax 52 CB (Agilent) (25 m × 0.25 mm × 1.2 µm); carrier gas: N_2<br>Split 150	135°C hold 4 min<br>20°C/min to 200°C hold 6.0 min<br>25°C/min to 250°C hold 1.0 min	3.56 min ethylbenzene<br>7.73 min n-octanol (IS)<br>9.72 min acetophenone<br>11.76 min 1-Phenylethanol
1-Chloro-4-ethylbenzene<br>1-(4-Chlorophenyl)ethanol<br>4'-Chloroacetophenone	CP-Sil 5 CB (Agilent) (25 m × 0.25 mm × 1.2 µm); carrier gas: N_2<br>Split 150	165°C hold 9 min<br>25°C/min to 345°C hold 1.0 min	3.74 min n-octanol (IS)<br>3.98 min 1-Chloro-4-ethylbenzene<br>7.02 min 4'-Chloroacetophenone<br>7.49 min 1-(4-Chlorophenyl)ethanol
1-Chloro-2-ethylbenzene<br>1-(2-Chlorophenyl)ethanol<br>2’-Chloroacetophenone	CP-Sil 5 CB (Agilent) (25 m × 0.25 mm × 1.2 µm); carrier gas: N_2<br>Split 150	110°C hold 3 min<br>20°C/min to 145°C hold 5.0 min<br>20°C/min to 170°C hold 3.0 min<br>25°C/min to 345°C hold 1.0 min	7.72 min 1-Chloro-2-ethylbenzene<br>7.90 min n-octanol (IS)<br>11.86 min 2’-Chloroacetophenone<br>12.67 min 1-(2-Chlorophenyl)ethanol
1-Chloro-3-ethylbenzene<br>1-(3-Chlorophenyl)ethanol<br>3’-Chloroacetophenone	CP-Wax 52 CB (Agilent) (50 m × 0.53 mm × 2.0 µm); carrier gas: N_2<br>Splitless	180°C hold 12 min<br>25°C/min to 250°C hold 1.0 min	1.58 min 1-Chloro-3-ethylbenzene<br>1.87 min n-octanol (IS)<br>4.97 min 3’-Chloroacetophenone<br>10.27 min 1-(3-Chlorophenyl)ethanol
1-Bromo-4-ethylbenzene<br>1-(4-Bromophenyl)ethanol<br>4'-Bromoacetophenone	CP-Wax 52 CB (Agilent) (25 m × 0.25 mm × 1.2 µm); carrier gas: N_2<br>Split 150	160°C hold 11 min<br>20°C/min to 220°C hold 2.0 min<br>25°C/min to 250°C hold 1.0 min	2.93 min 1-Bromo-4-ethylbenzene<br>6.49 min n-octanol (IS)<br>9.90 min 4'-Bromoacetophenone<br>14.37 min 1-(4-Bromophenyl)ethanol
1-Ethyl-4-fluorobenzene<br>1-(4-Fluorophenyl)ethanol<br>4-Fluoroacetophenone	CP-Sil 5 CB (Agilent) (25 m × 0.25 mm × 1.2 µm); carrier gas: N_2<br>Split 150	170°C hold 10 min<br>25°C/min to 345°C hold 1.0 min	3.36 min n-octanol (IS)<br>4.68 min 1-Ethyl-4-fluorobenzene<br>8.49 min 4-Fluoroacetophenone<br>9.06 min 1-(4-Fluorophenyl)ethanol
1-Ethyl-4-iodobenzene<br>1-(4-Iodophenyl)ethanol<br>4-Iodoacetophenone	CP-Sil 5 CB (Agilent) (25 m × 0.25 mm × 1.2 µm); carrier gas: N_2<br>Split 150	190°C hold 15 min<br>25°C/min to 345°C hold 1.0 min	2.75 min n-octanol (IS)<br>4.82 min 1-Ethyl-4-iodobenzene<br>8.41 min 4-Iodoacetophenone<br>8.75 min 1-(4-Iodophenyl)ethanol
4-Ethylnitrobenzene<br>1-(4-Nitrophenyl)ethanol<br>4-Nitroacetophenone	CP-Sil 5 CB (Agilent) (25 m × 0.25 mm × 1.2 µm); carrier gas: N_2<br>Split 150	170°C hold 22 min<br>25°C/min to 345°C hold 1.0 min	3.48 min n-octanol (IS)<br>8.50 min 4-Ethylnitrobenzene<br>13.32 min 4-Nitroacetophenone<br>18.14 min 1-(4-Nitrophenyl)ethanol
4-Ethylanisole<br>1-(4-Methoxyphenyl)ethanol<br>4'-Methoxyacetophenone	CP-Sil 5 CB (Agilent) (25 m × 0.25 mm × 1.2 µm); carrier gas: N_2<br>Split 150	160°C hold 6 min<br>20°C/min to 205°C hold 1.5 min<br>25°C/min to 345°C hold 1.0 min	4.05 min n-octanol (IS)<br>4.99 min 4-Ethylanisole<br>8.35 min 1-(4-Methoxyphenyl)ethanol<br>9.12 min 4'-Methoxyacetophenone
2-Ethylanisole<br>1-(4-Methoxyphenyl)ethanol<br>2-Methoxyacetophenone	CP-Sil 5 CB (Agilent) (25 m × 0.25 mm × 1.2 µm); carrier gas: N_2; Split 150	180°C hold 10 min<br>25°C/min to 345°C hold 1.0 min	3.06 min n-octanol (IS)<br>3.36 min 2-Ethylanisole<br>5.74 min 1-(2-Methoxyphenyl)ethanol<br>5.99 min 2-Methoxyacetophenone
Isobutylbenzene<br>2-Methyl-1-phenyl-1-propanol<br>Isobutyrophenone	CP-Sil 5 CB (Agilent) (25 m × 0.25 mm × 1.2 µm); carrier gas: N_2; Split 150	100°C hold 3 min<br>25°C/min to 345°C hold 1.0 min	9.38 min n-octanol (IS)<br>9.68 min Isobutylbenzene

Table S2. GC analytical data with chiral columns

Compound	GC column	Oven temperature program	Retention time
Ethylbenzene<br>1-Phenylethanol<br>Acetophenone	CP-Chirasil-Dex-CB (Agilent) (25 m × 0.32 mm × 0.25 µm); carrier gas: He<br>Split 150	100°C hold 16 min<br>20°C/min to 140°C hold 3.0 min<br>25°C/min to 225°C hold 1.0 min	3.49 min Ethylbenzene<br>10.07 min Acetophenone<br>14.16 min n-octanol (IS)<br>18.65 min (*R*)-1-Phenylethanol<br>19.10 min (*S*)-1-Phenylethanol

1-Chloro-4-ethylbenzene<br>1-(4-Chlorophenyl)ethanol<br>4'-Chloroacetophenone	CP-Chirasil-Dex-CB (Agilent) (25 m × 0.32 mm × 0.25 µm); carrier gas: He Split 150	150°C hold 10 min<br>25°C/min to 225°C hold 1.0 min	2.70 min 1-Chloro-4-ethylbenzene<br>2.95 min n-octanol (IS)<br>4.52 min 4'-Chloroacetophenone<br>7.54 min (*R*)-1-(4-Chlorophenyl)ethanol<br>8.08 min (*S*)-1-(4-Chlorophenyl)ethanol
1-Chloro-2-ethylbenzene<br>1-(2-Chlorophenyl)ethanol<br>2'-Chloroacetophenone	CP-Chirasil-Dex-CB (Agilent) (25 m × 0.32 mm × 0.25 µm); carrier gas: He Split 150	150°C hold 10 min<br>25°C/min to 225°C hold 1.0 min	2,73 min 1-Chloro-2-ethylbenzene<br>2.96 min n-octanol (IS)<br>3.76 min 2'-Chloroacetophenone<br>6.89 min (*R*)-1-(2-Chlorophenyl)ethanol<br>7.73 min (*S*)-1-(2-Chlorophenyl)ethanol
1-Chloro-3-ethylbenzene<br>1-(3-Chlorophenyl)ethanol<br>3'-Chloroacetophenone	CP-Chirasil-Dex-CB (Agilent) (25 m × 0.32 mm × 0.25 µm); carrier gas: He Split 150	150°C hold 10 min<br>25°C/min to 225°C hold 1.0 min	2.77 min 1-Chloro-3-ethylbenzene<br>2.96 min n-octanol (IS)<br>4.12 min 3'-Chloroacetophenone<br>7.31 min (*R*)-1-(3-Chlorophenyl)ethanol<br>7.68 min (*S*)-1-(3-Chlorophenyl)ethanol
1-Bromo-4-ethylbenzene<br>1-(4-Bromophenyl)ethanol<br>4'-Bromoacetophenone	CP-Chirasil-Dex-CB (Agilent) (25 m × 0.32 mm × 0.25 µm); carrier gas: He Split 150	135°C hold 8 min<br>25°C/min to 225°C hold 1.0 min	2.37 min 1-Bromo-4-ethylbenzene<br>3.66 min 4'-Bromoacetophenone<br>3.92 min n-octanol (IS)<br>5.99 min (*R*)-1-(4-Bromophenyl)ethanol<br>6.41 min (*S*)-1-(4-Bromophenyl)ethanol
1-Ethyl-4-fluorobenzene<br>1-(4-Fluorophenyl)ethanol<br>4-Fluoroacetophenone	CP-Chirasil-Dex-CB (Agilent) (25 m × 0.32 mm × 0.25 µm); carrier gas: He Split 150	160°C hold 10 min<br>25°C/min to 225°C hold 1.0 min	2.72 min n-octanol (IS)<br>2.97 min 1-Ethyl-4-fluorobenzene<br>5.02 min 4-Fluoroacetophenone<br>8.03 min (*R*)-1-(4-Fluorophenyl)ethanol<br>8.48 min (*S*)-1-(4-Fluorophenyl)ethanol
1-Ethyl-4-iodobenzene<br>1-(4-Iodophenyl)ethanol<br>4-Iodoacetophenone	CP-Chirasil-Dex-CB (Agilent) (25 m × 0.32 mm × 0.25 µm); carrier gas: He Split 150	175°C hold 8 min<br>25°C/min to 225°C hold 1.0 min	2.31 min n-octanol (IS)<br>2.89 min 1-Ethyl-4-iodobenzene<br>5.00 min 4-Iodoacetophenone<br>6.848 min (*R*)-1-(4-Iodophenyl)ethanol<br>7.08 min (*S*)-1-(4-Iodophenyl)ethanol
4-Ethylnitrobenzene<br>1-(4-Nitrophenyl)ethanol<br>4-Nitroacetophenone	CP-Chirasil-Dex-CB (Agilent) (25 m × 0.32 mm × 0.25 µm); carrier gas: He Split 150	180°C hold 13 min<br>25°C/min to 225°C hold 1.0 min	2.31 min n-octanol (IS)<br>3.31 min 4-Ethylnitrobenzene<br>4.97 min 4-Nitroacetophenone<br>9.88 min (*R*)-1-(4-nitrophenyl)ethanol<br>10.54 min (*S*)-1-(4-nitrophenyl)ethanol
4-Ethylanisole<br>1-(4-Methoxyphenyl)ethanol<br>4'-Methoxyacetophenone	CP-Chirasil-Dex-CB (Agilent) (25 m × 0.32 mm × 0.25 µm); carrier gas: He Split 150	110°C hold 3 min<br>20°C/min to 130°C hold 3.0 min<br>20°C/min to 170°C hold 3.0 min<br>25°C/min to 225°C hold 1.0 min	5.48 min 4-Ethylanisole<br>6.25 min n-octanol (IS)<br>10.39 min 4'-Methoxyacetophenone<br>11.23 min (*R*)-1-(4-Methoxyphenyl)ethanol<br>11.38 min (*S*)-1-(4-Methoxyphenyl)ethanol
2-Ethylanisole<br>1-(2-Methoxyphenyl)ethanol<br>2-Methoxyacetophenone	CP-Chirasil-Dex-CB (Agilent) (25 m × 0.32 mm × 0.25 µm); carrier gas: He; Split 150	110°C hold 3 min<br>20°C/min to 130°C hold 3.0 min<br>20°C/min to 180°C hold 5.0 min<br>25°C/min to 225°C hold 1.0 min	5.22 min 2-Ethylanisole<br>6.57 min n-octanol (IS)<br>10.83 min 2-Methoxyacetophenone<br>12.95 min (*R*)-1-(2-Methoxyphenyl)ethanol

5. GC chromatograms

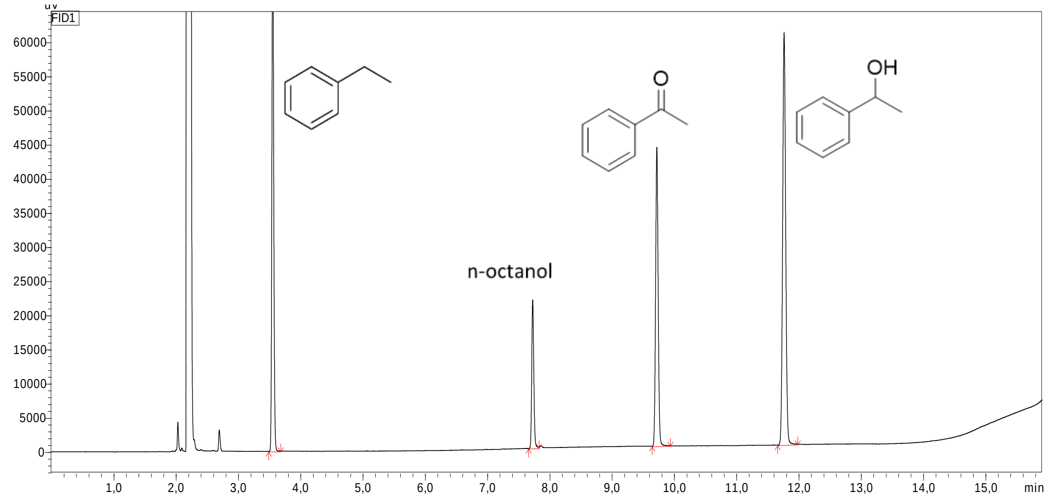

Figure S3. GC chromatogram of ethylbenzene, 1-phenylethanol, acetophenone commercial compounds.

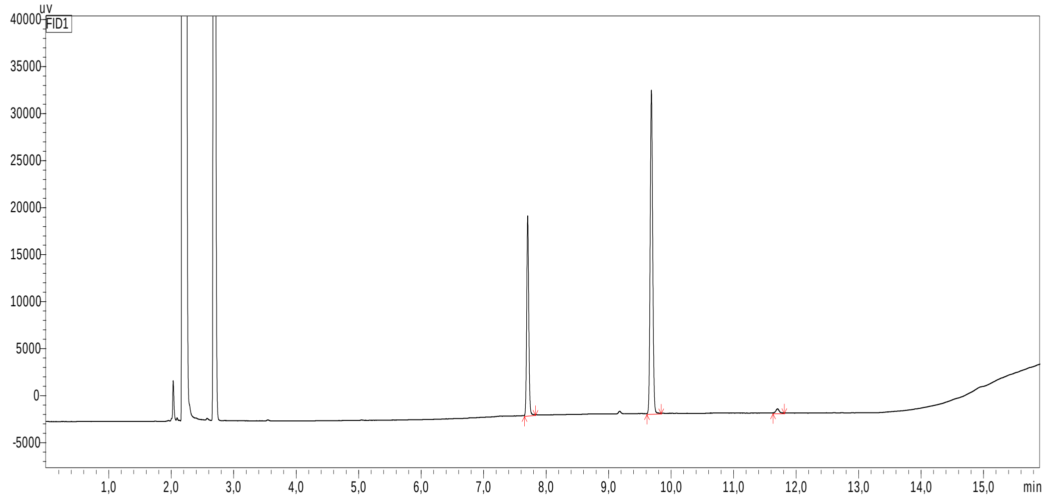

Figure S4. GC chromatogram of ethylbenzene reaction products with r*Aae*UPO.

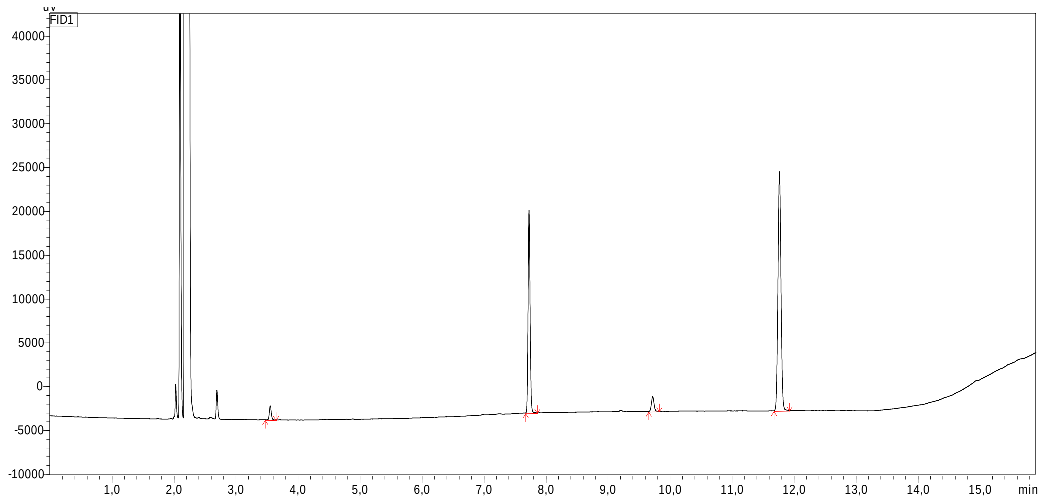

Figure S5. GC chromatogram of ethylbenzene reaction products with r*Aae*UPO and ADH-A, one-pot two-step cascade.

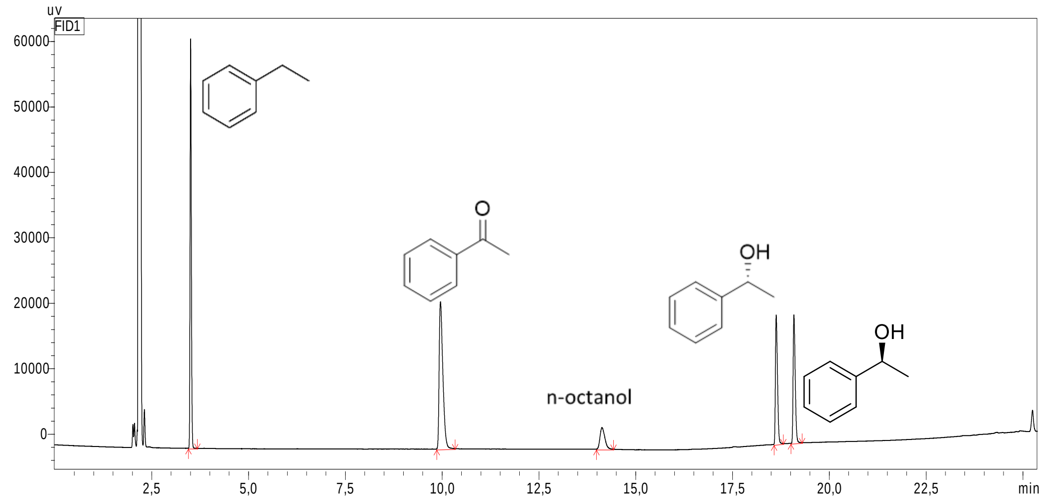

Figure S6. Chiral GC chromatogram of ethylbenzene, 1-phenylethanol, acetophenone commercial compounds.

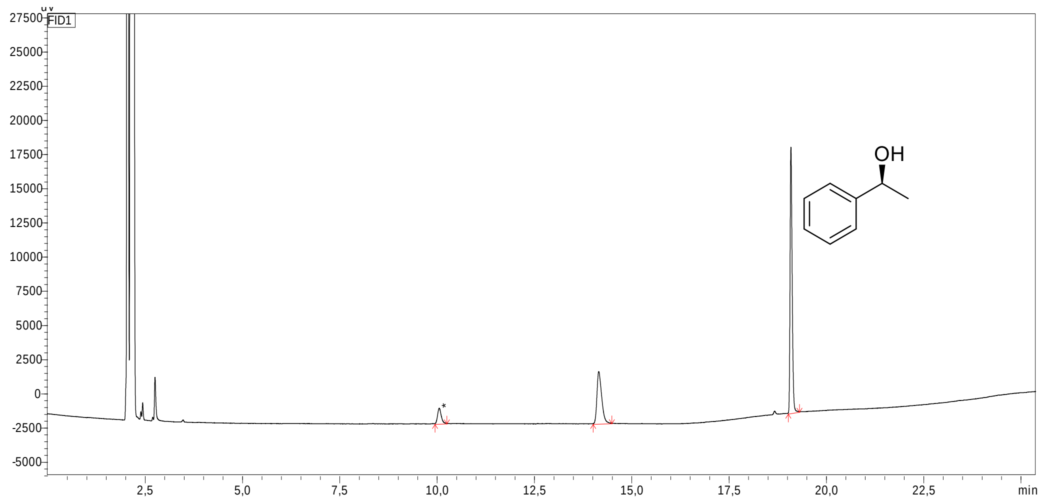

Figure S7. Chiral GC chromatogram of ethylbenzene reaction products with r*Aae*UPO and ADH-A, one-pot two-step cascade.

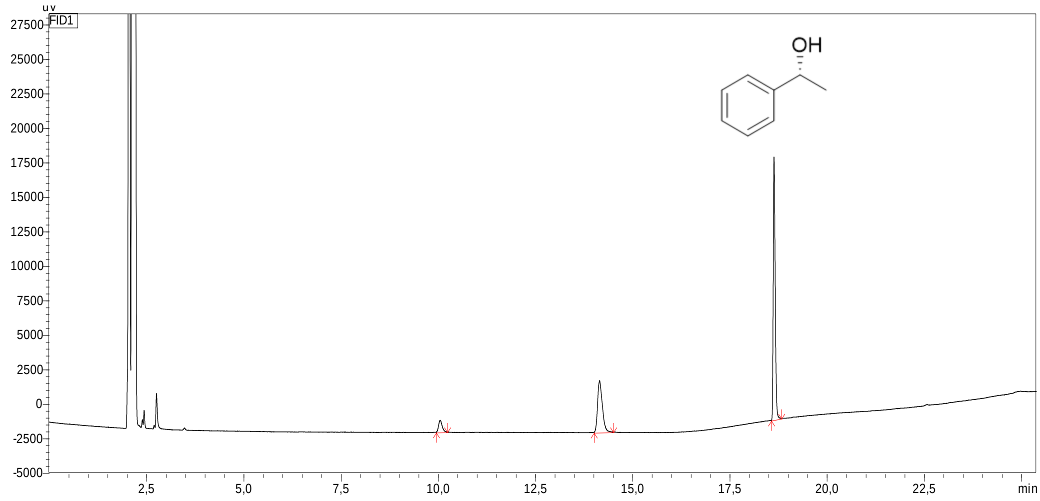

Figure S8. Chiral GC chromatogram of ethylbenzene reaction products with r*Aae*UPO and *Lk*ADH, one-pot two-step cascade.

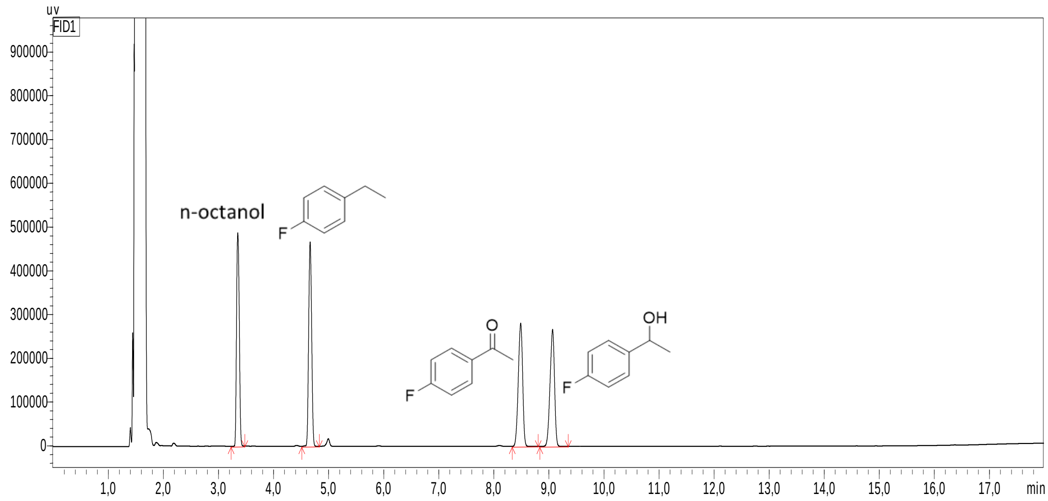

Figure S9. GC chromatogram of 1-ethyl-4-fluorobenzene, 1-(4-fluorophenyl)ethanol, 4-fluoroacetophenone commercial compounds.

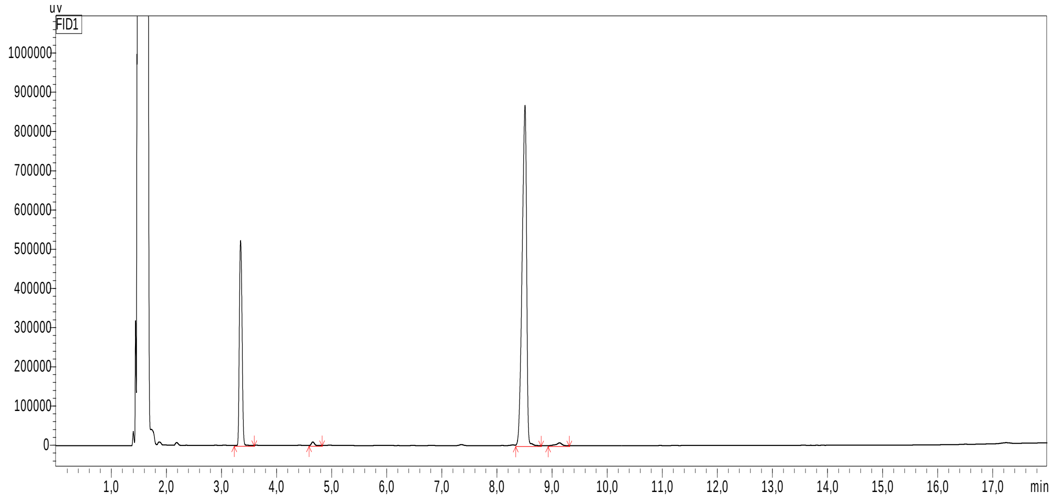

Figure S10. GC chromatogram of 1-ethyl-4-fluorobenzene reaction products with r*Aae*UPO

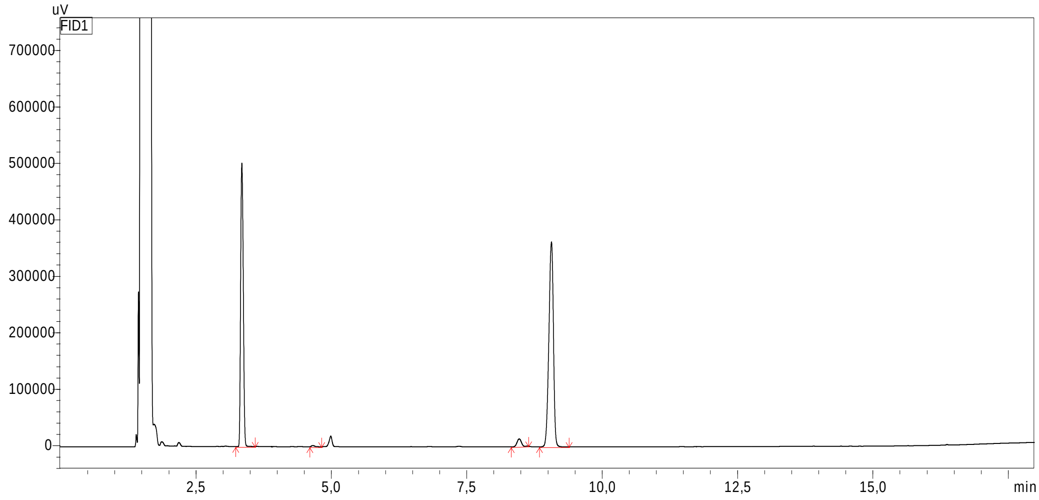

Figure S11. GC chromatogram of 1-ethyl-4-fluorobenzene reaction products with r*Aae*UPO and ADH-A, one-pot two-step cascade.

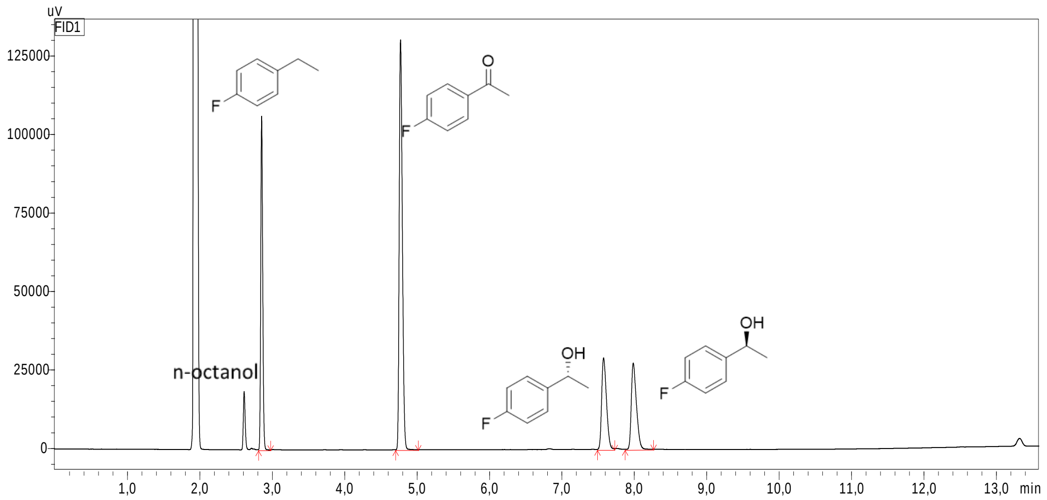

Figure S12. Chiral GC chromatogram of 1-ethyl-4-fluorobenzene, 1-(4-fluorophenyl)ethanol, 4-fluoroacetophenone commercial compounds.

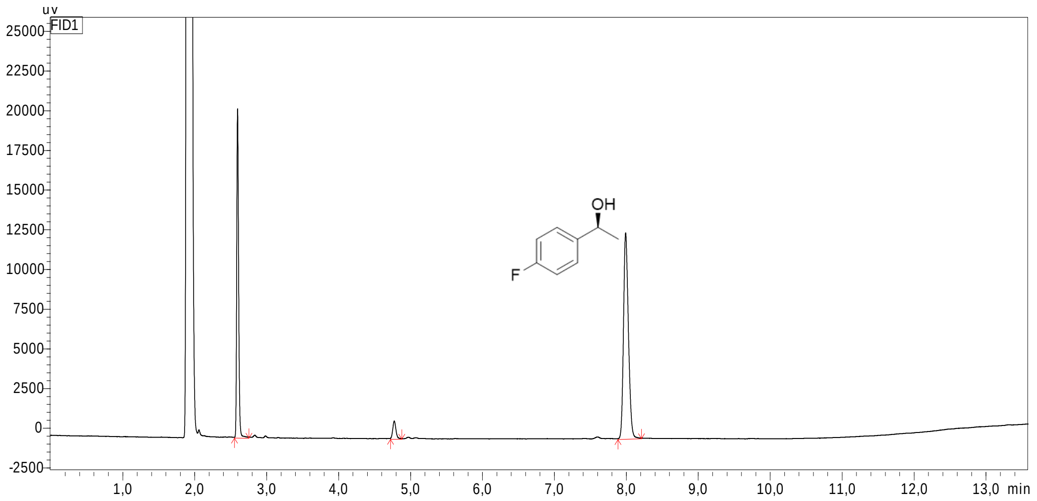

Figure S13. Chiral GC chromatogram of 1-ethyl-4-fluorobenzene reaction products with r*Aae*UPO and ADH-A, one-pot two-step cascade.

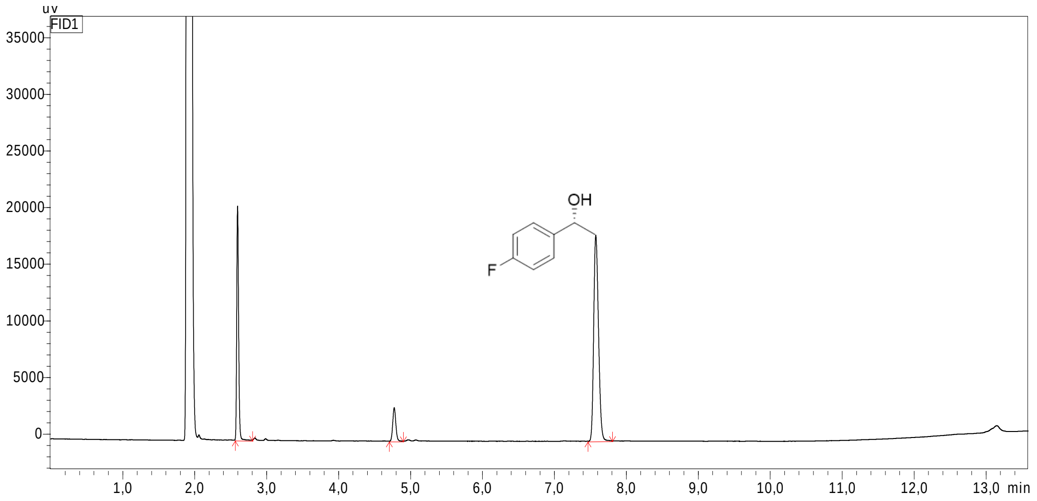

Figure S14. Chiral GC chromatogram of 1-ethyl-4-fluorobenzene reaction products with r*Aae*UPO and *Lk*ADH, one-pot two-step cascade.

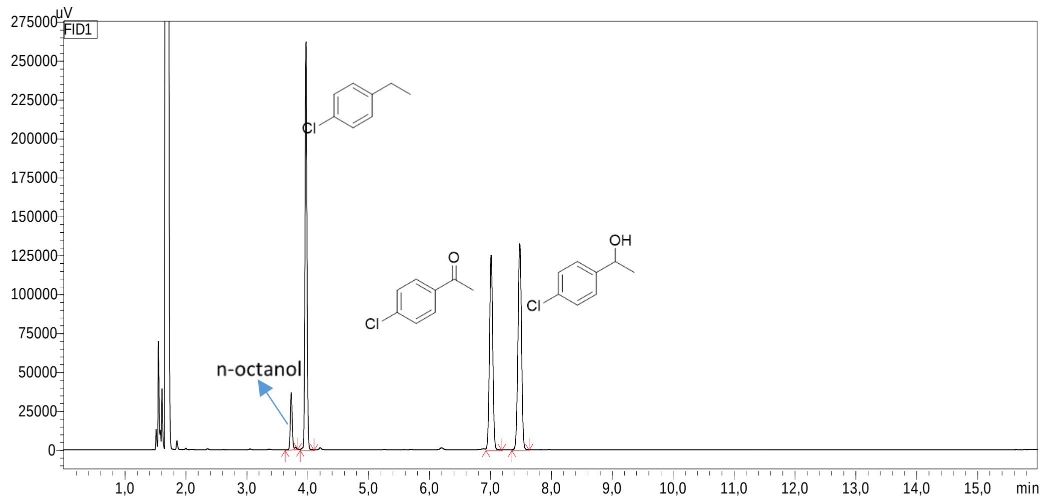

Figure S15. GC chromatogram of 1-chloro-4-ethylbenzene, 1-(4-chlorophenyl)ethanol, 4'-chloroacetophenone commercial compounds.

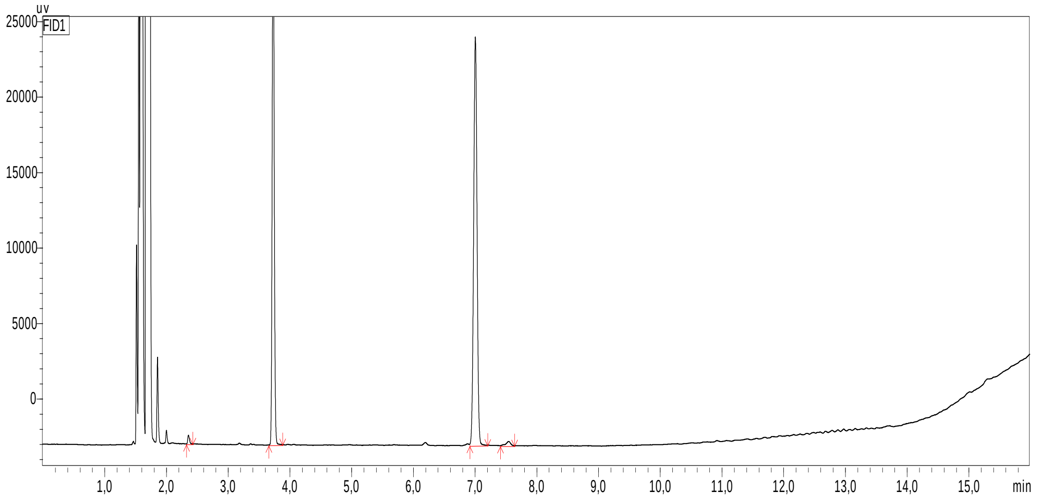

Figure S16. GC chromatogram of 1-chloro-4-ethylbenzene reaction products with r*Aae*UPO.

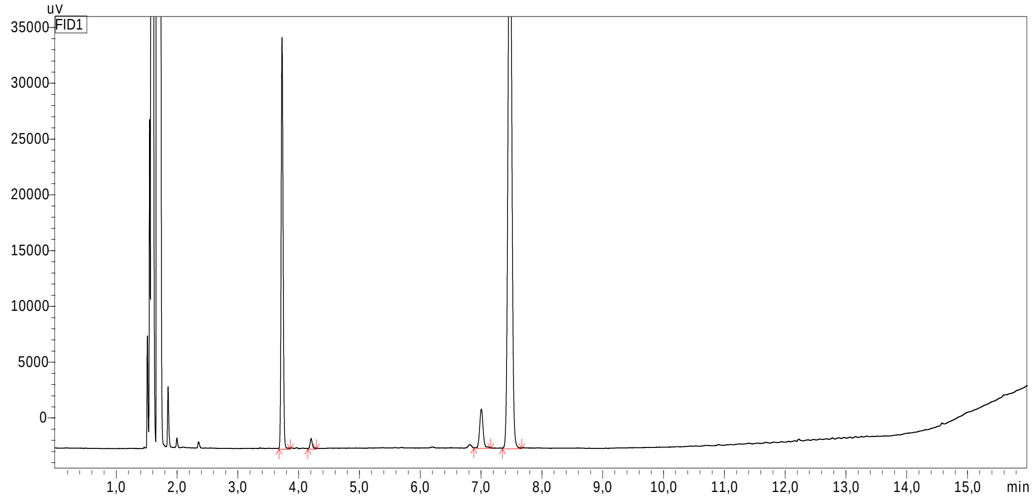

Figure S17. GC chromatogram of 1-chloro-4-ethylbenzene reaction products with r*Aae*UPO and ADH-A, one-pot two-step cascade.

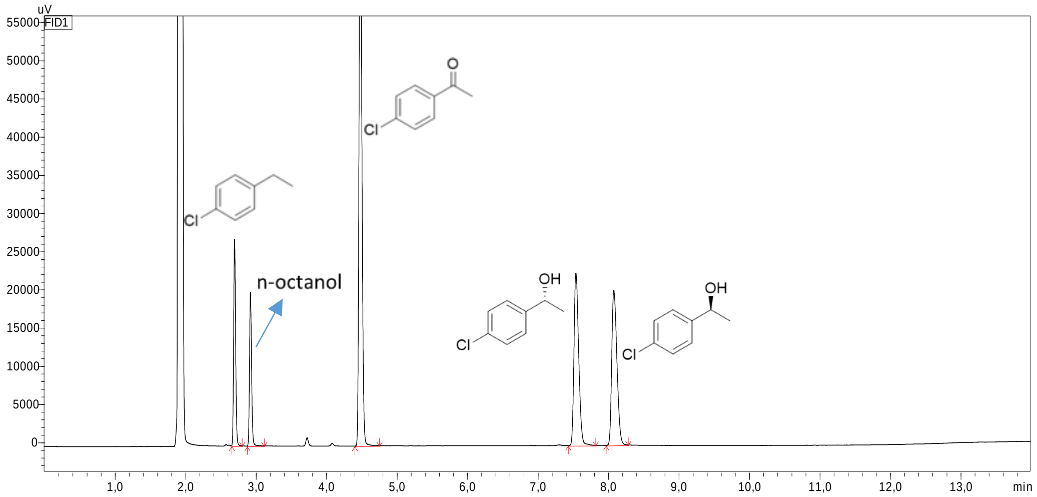

Figure S18. Chiral GC chromatogram of 1-chloro-4-ethylbenzene, 1-(4-chlorophenyl)ethanol, 4-chloroacetophenone commercial compounds.

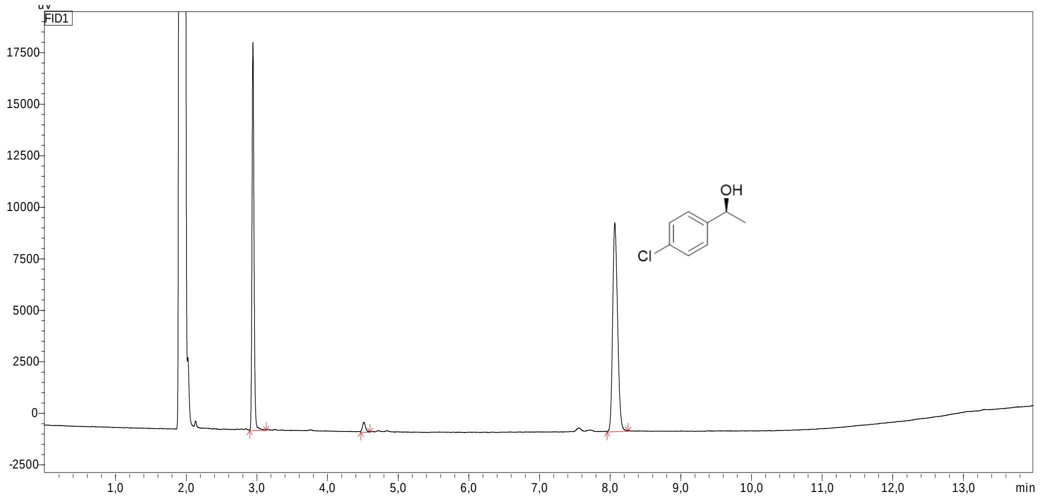

Figure S19. Chiral GC chromatogram of 1-chloro-4-ethylbenzene reaction products with r*Aae*UPO and ADH-A, one-pot two-step cascade.

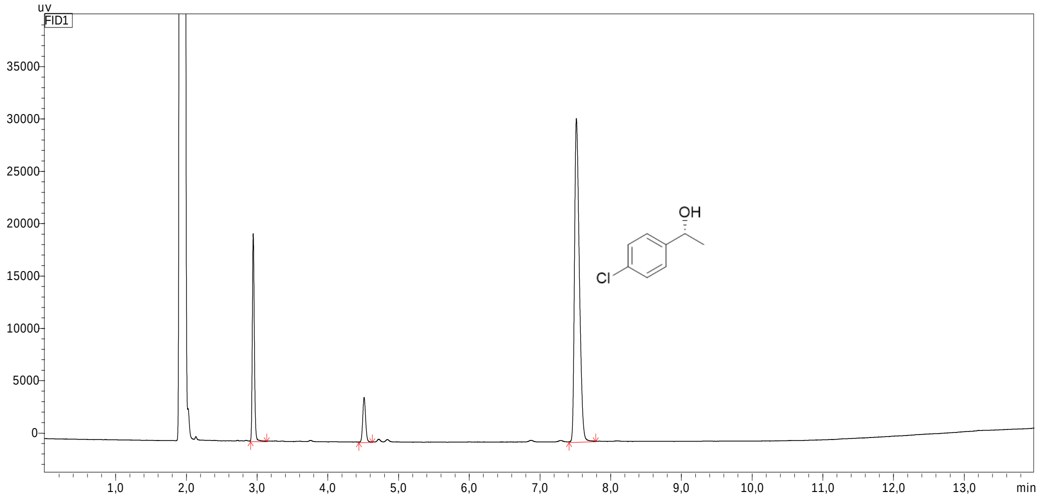

Figure S20. Chiral GC chromatogram of 1-chloro-4-ethylbenzene reaction products with r*Aae*UPO and *Lk*ADH, one-pot two-step cascade.

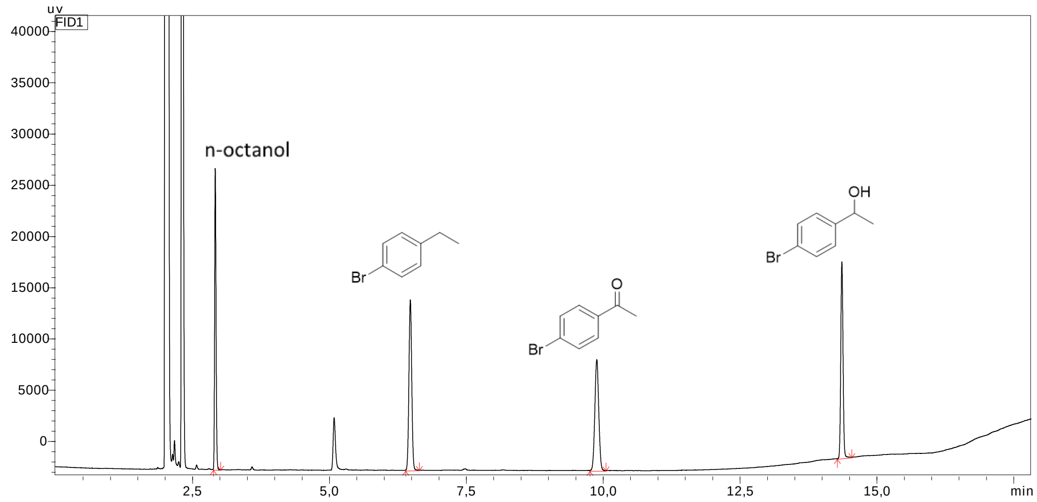

Figure S21. GC chromatogram of 1-bromo-4-ethylbenzene, 1-(4-bromophenyl)ethanol, 4-bromoacetophenone commercial compounds.

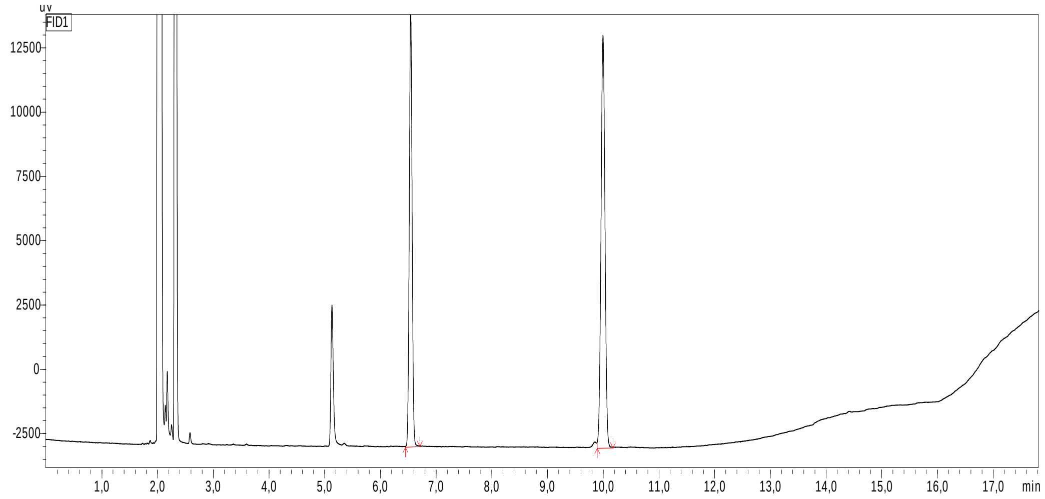

Figure S22. GC chromatogram of 1-bromo-4-ethylbenzene reaction products with r*Aae*UPO.

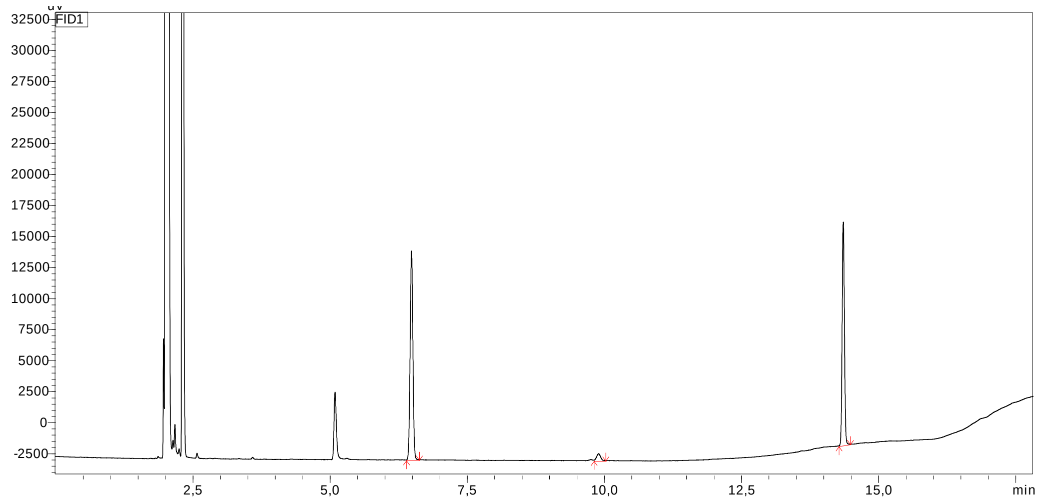

Figure S23. GC chromatogram of 1-bromo-4-ethylbenzene reaction products with r*Aae*UPO and ADH-A, one-pot two-step cascade.

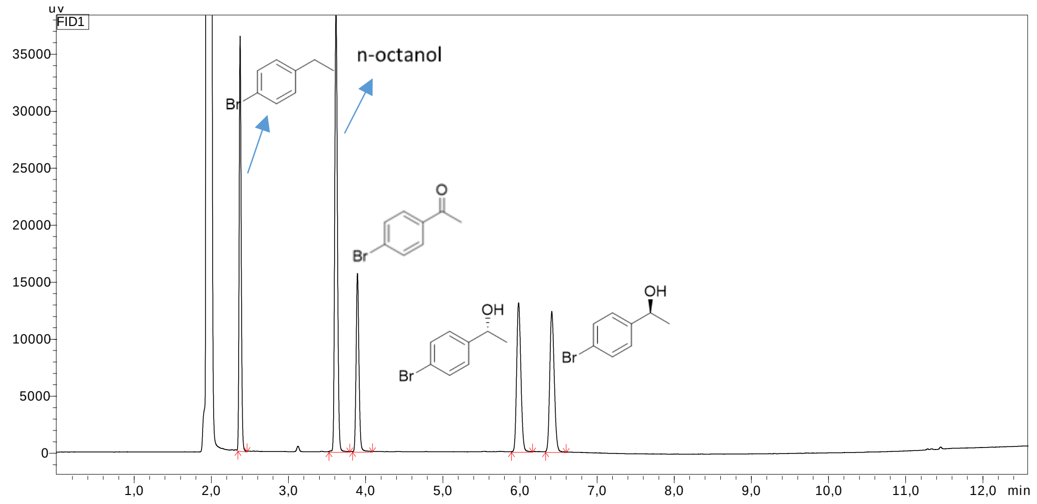

Figure S24. Chiral GC chromatogram of 1-bromo-4-ethylbenzene, 1-(4-bromophenyl)ethanol, 4-bromoacetophenone commercial compounds.

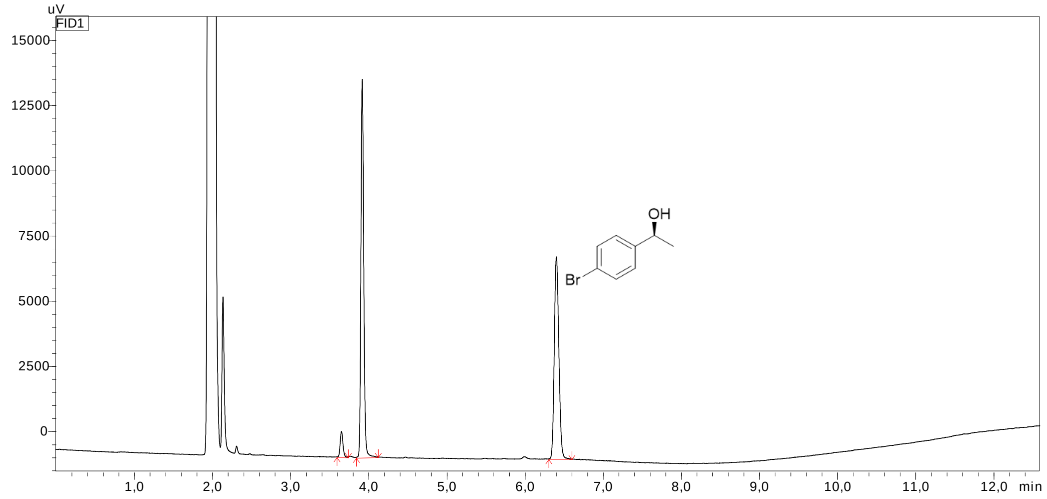

Figure 25. Chiral GC chromatogram of 1-bromo-4-ethylbenzene reaction products with r*Aae*UPO and ADH-A, one-pot two-step cascade.

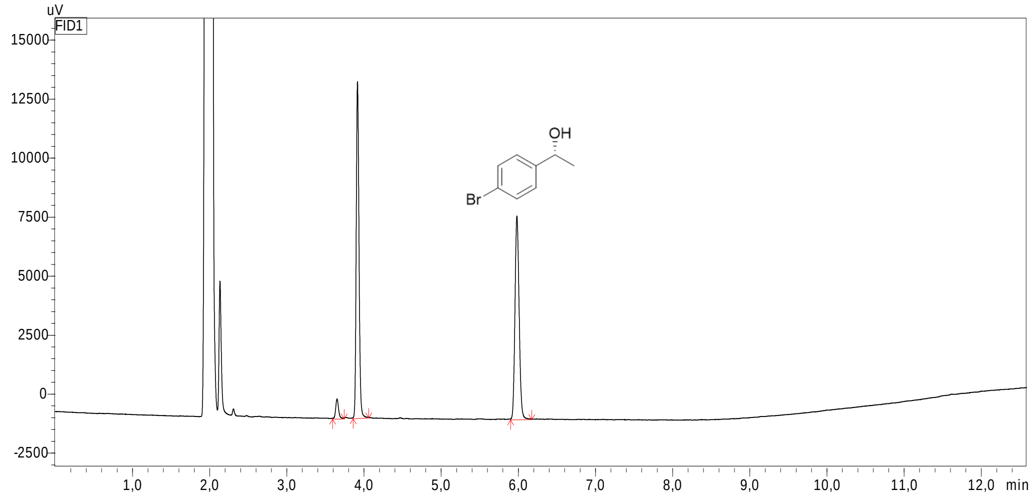

Figure S26. Chiral GC chromatogram of 1-bromo-4-ethylbenzene reaction products with r*Aae*UPO and *Lk*ADH one-pot two-step cascade.

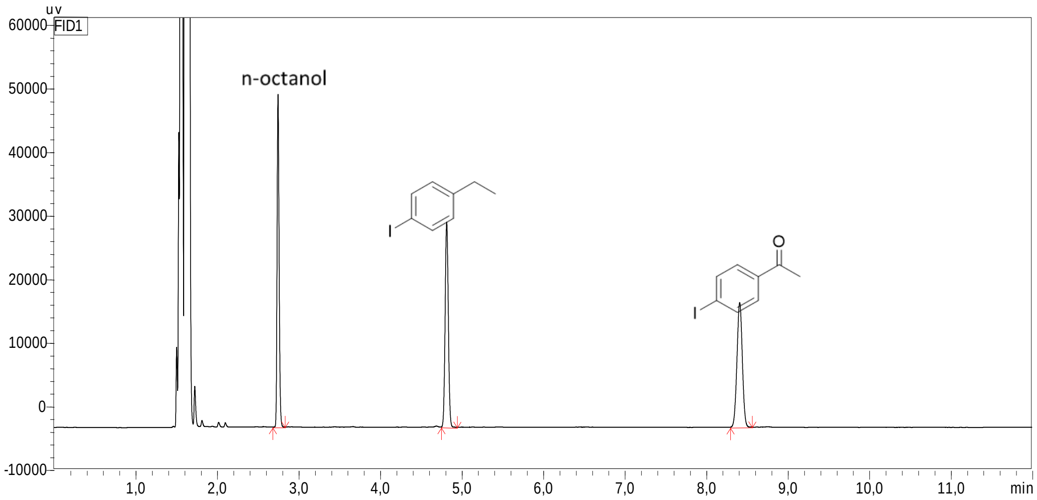

Figure S27. GC chromatogram of 1-ethyl-4-iodobenzene, 4-iodoacetophenone commercial compounds. (1-(4-Iodophenyl)ethanol was identified by GC-MS, Figure S63)

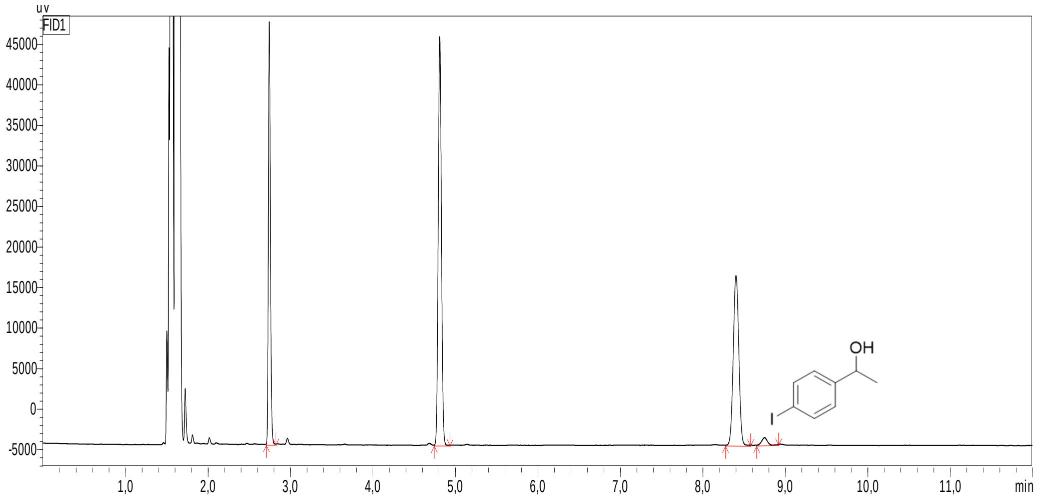

Figure S28. GC chromatogram of 1-ethyl-4-iodobenzene reaction product with r*Aae*UPO. (1-(4-iodophenyl)ethanol was identified by GC-MS, Figure S63)

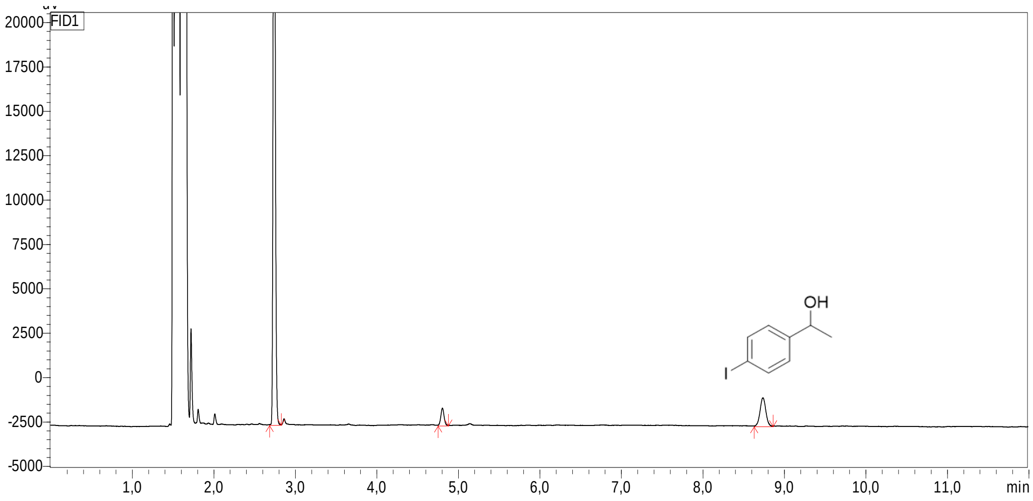

Figure S29. GC chromatogram of 1-ethyl-4-iodobenzene reaction products with r*Aae*UPO and ADH-A, one-pot two-step cascade.

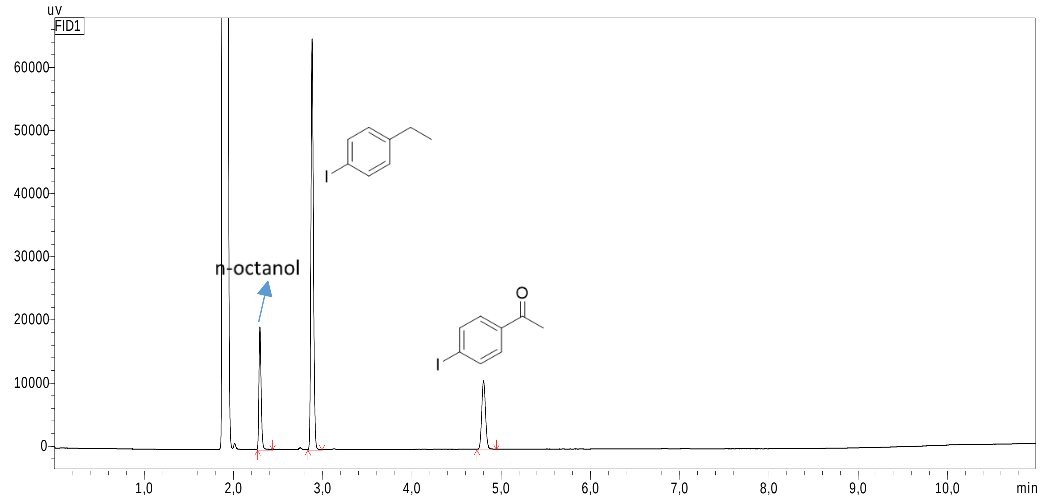

Figure S30. Chiral GC chromatogram of 1-ethyl-4-Iodobenzene, 4-iodoacetophenone commercial compounds. (1-(4-iodophenyl)ethanol was identified by GC-MS, Figure S63)

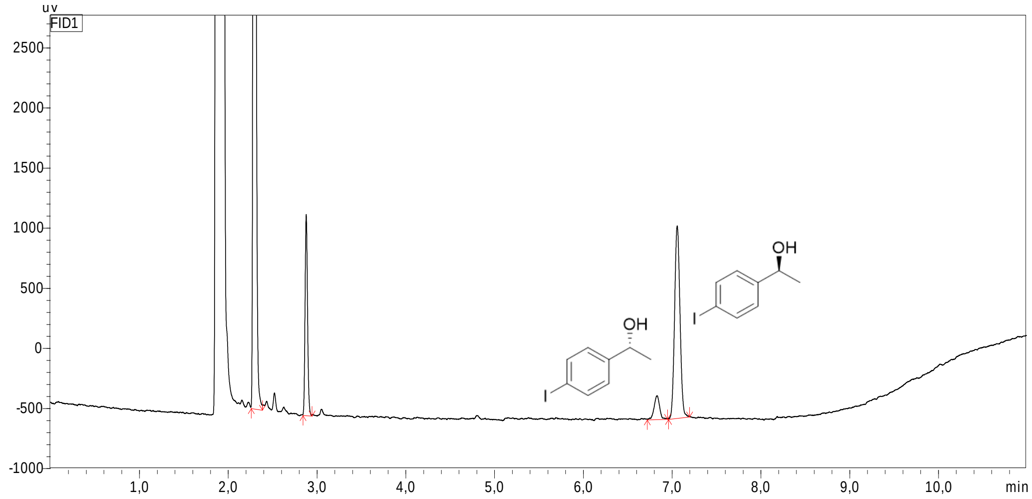

Figure S31. Chiral GC chromatogram of 1-ethyl-4-Iodobenzene reaction products with r*Aae*UPO and ADH-A, one-pot two-step cascade.

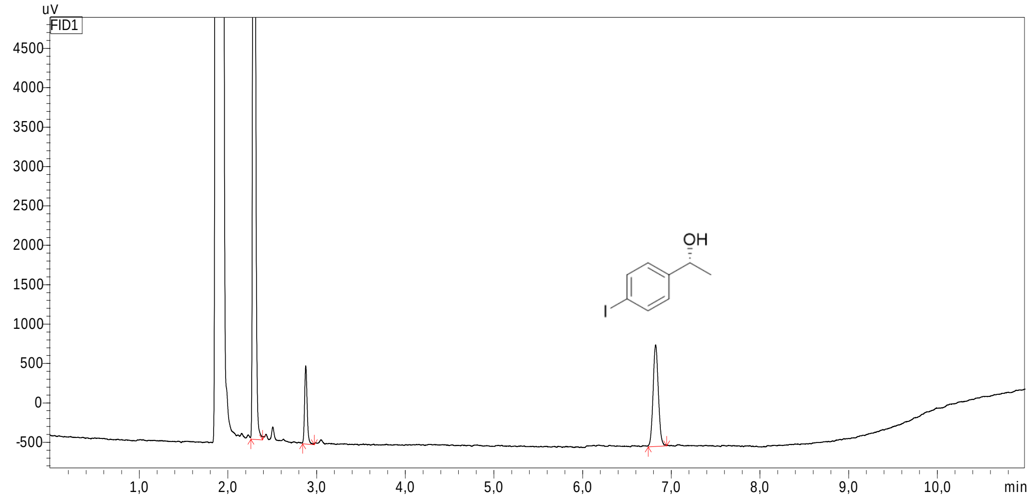

Figure S32. Chiral GC chromatogram of 1-ethyl-4-iodobenzene reaction products with r*Aae*UPO and *Lk*ADH, one-pot two-step cascade.

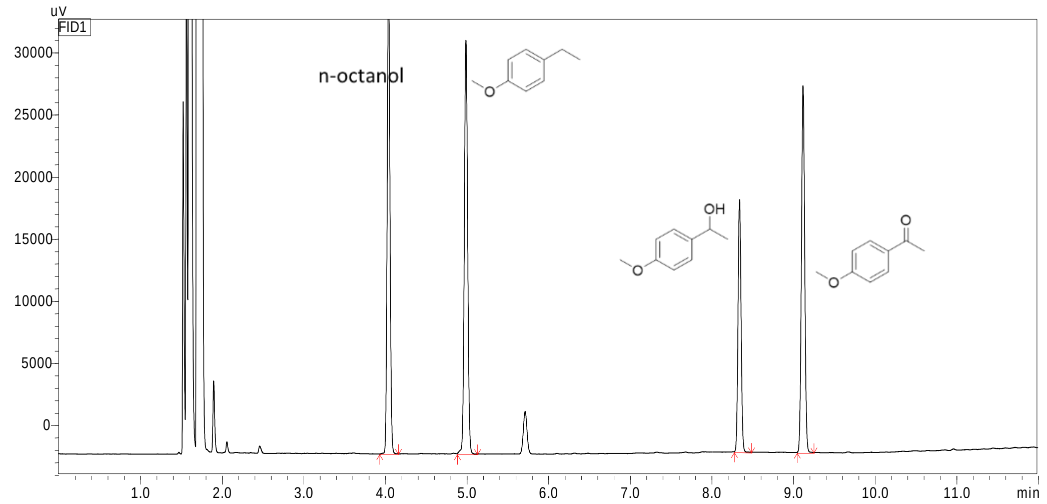

Figure S33. GC chromatogram of 4-ethylanisole, 1-(4-methoxyphenyl)ethanol, 4-methoxyacetophenone commercial compounds.

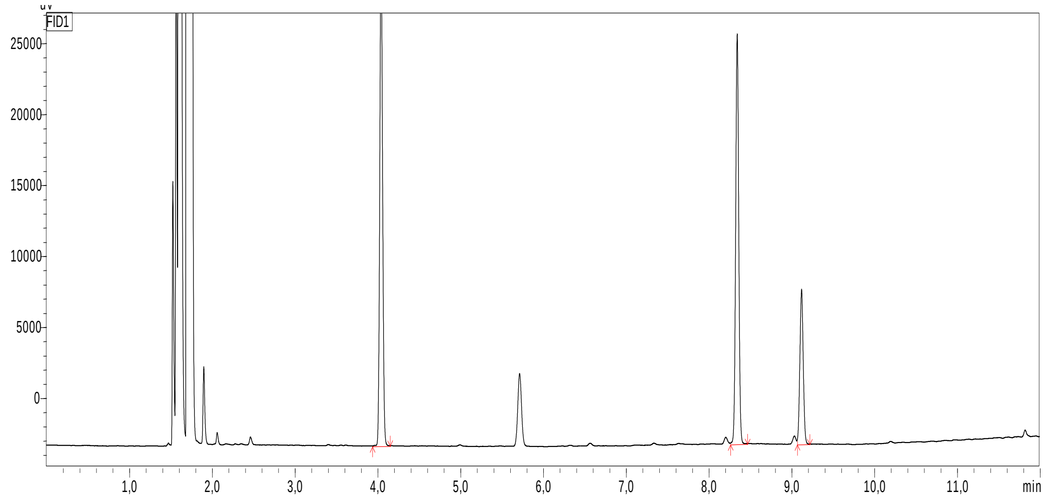

Figure S34. GC chromatogram of 4-ethylanisole reaction products with r*Aae*UPO.

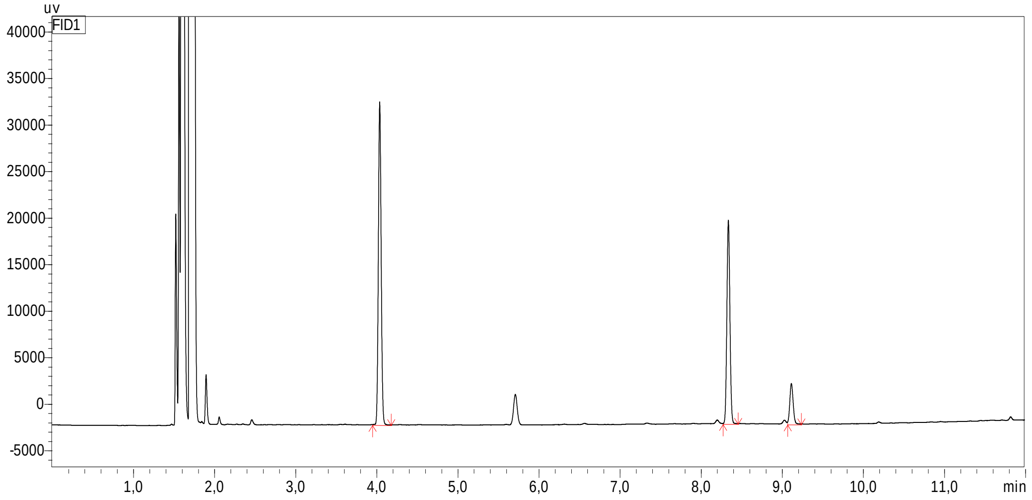

Figure S35. GC chromatogram of 4-ethylanisole reaction products with r*Aae*UPO and ADH-A, one-pot two-step cascade.

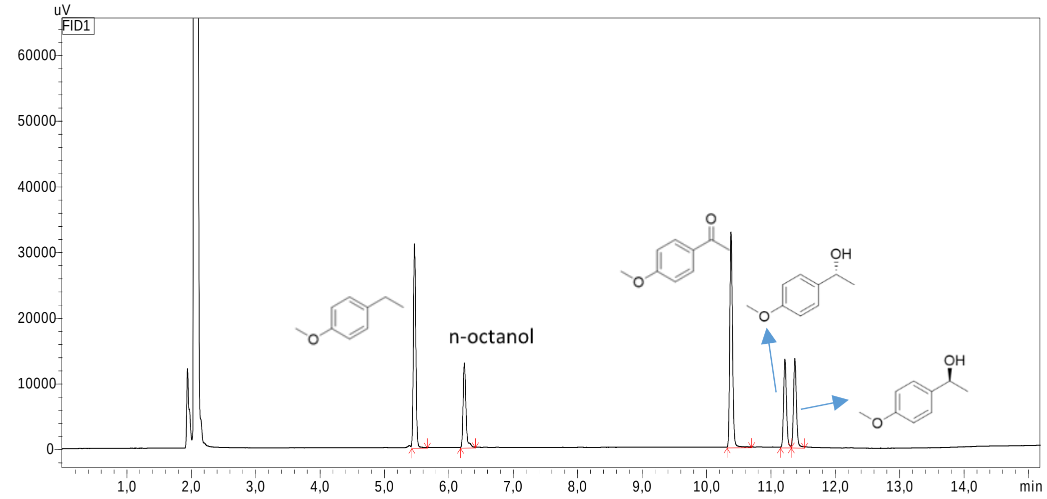

Figure S36. Chiral GC chromatogram of 4-ethylanisole, 4-methoxyacetophenone, 1-(4-methoxyphenyl)ethanol commercial compounds.

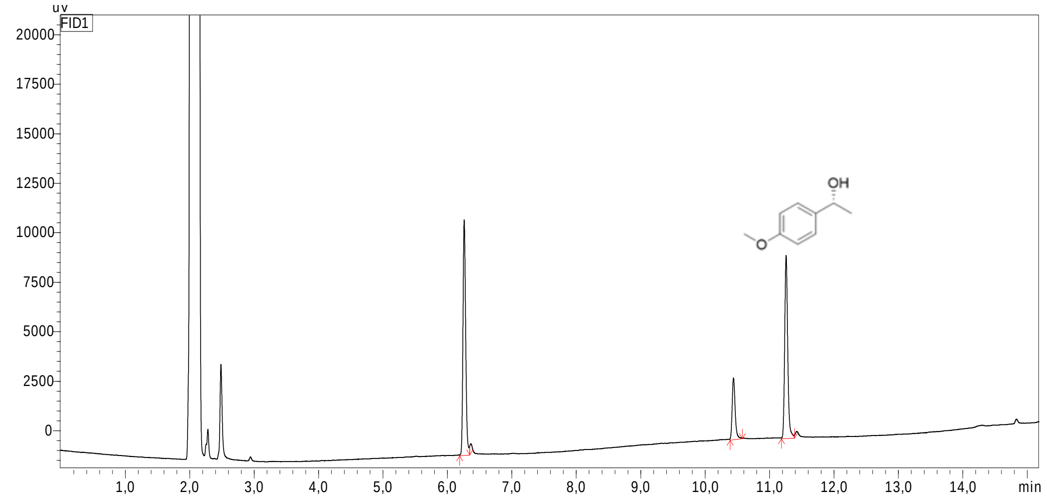

Figure S37. Chiral GC chromatogram of 4-ethylanisole reaction products with r*Aae*UPO and ADH-A, one-pot two-step cascade.

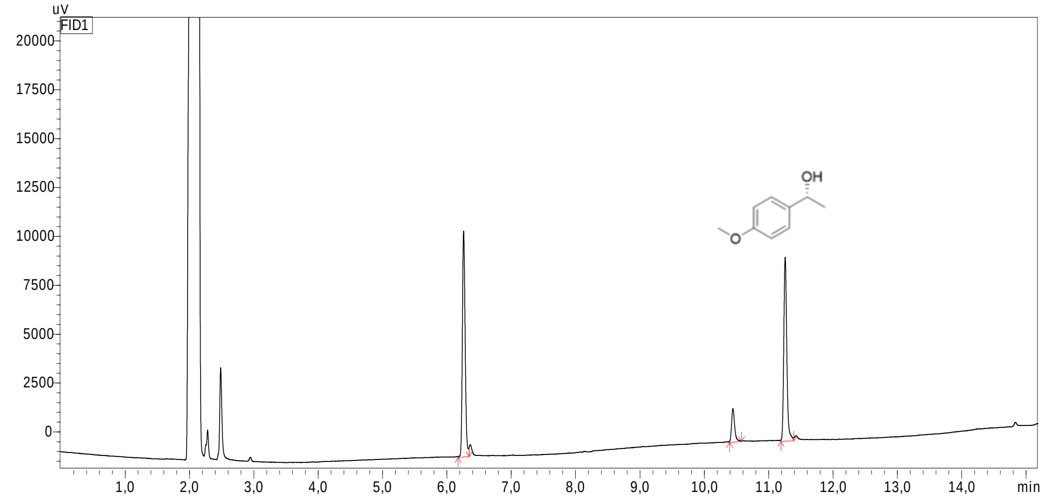

Figure S38. Chiral GC chromatogram of 4-ethylanisole reaction products with r*Aae*UPO and *Lk*ADH, one-pot two-step cascade.

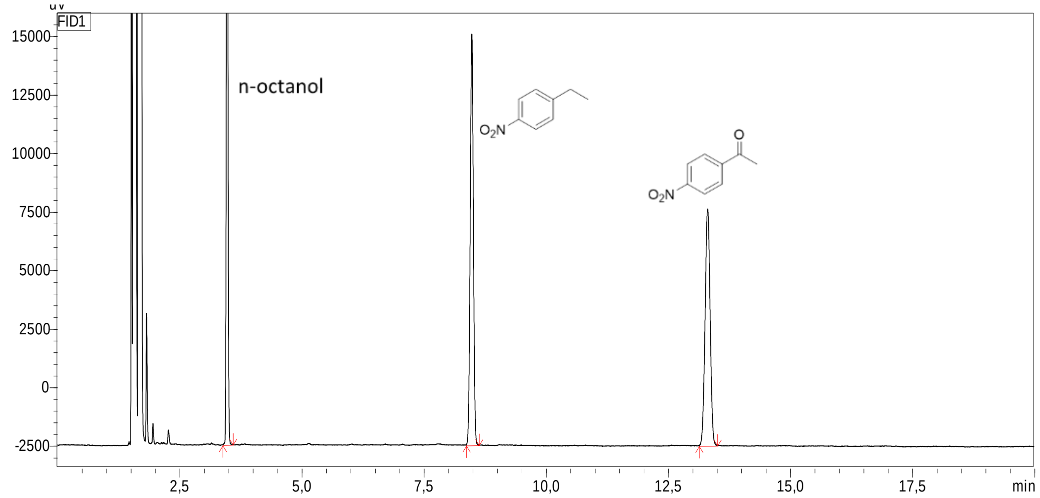

Figure S39. GC chromatogram of 4-ethylnitrobenzene, 4-nitroacetophenone commercial compounds. (1-(4-nitrophenyl)ethanol was identified by GC-MS, Figure S64)

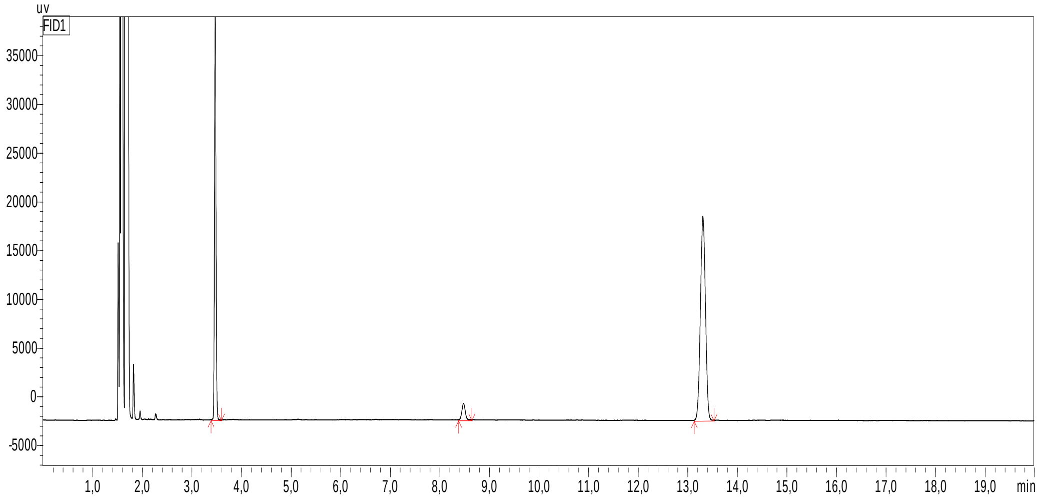

Figure S40. GC chromatogram of 4-ethylnitrobenzene reaction products with r*Aae*UPO. (1-(4-nitrophenyl)ethanol was identified by GC-MS, Figure S64)

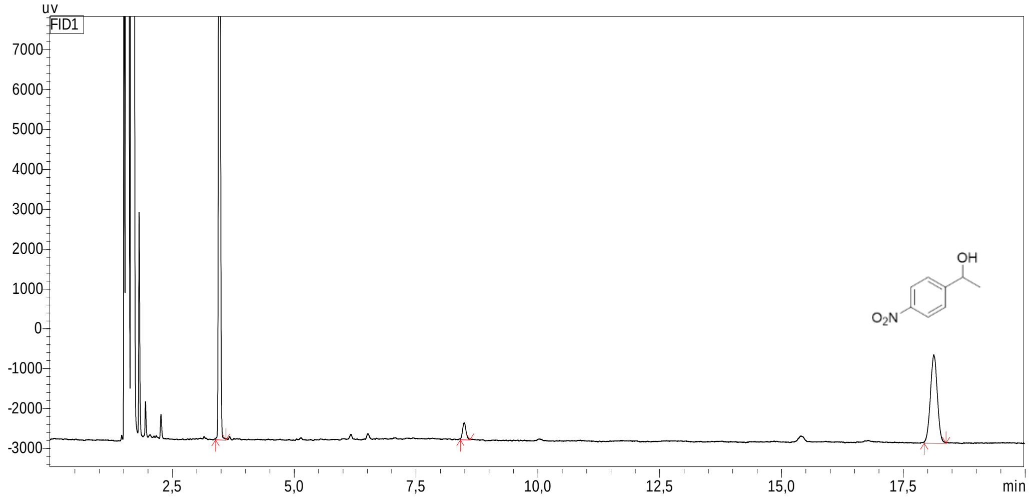

Figure S41. GC chromatogram of 4-ethylnitrobenzene reaction products with r*Aae*UPO and ADH-A, one-pot two-step cascade.

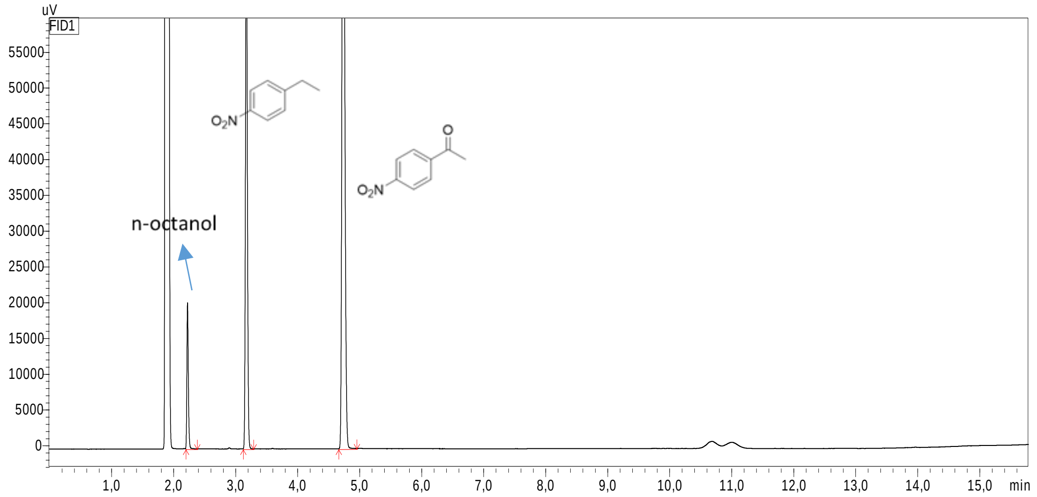

Figure S42. Chiral GC chromatogram of 4-ethylnitrobenzene, 4-nitroacetophenone commercial compounds. (1-(4-nitrophenyl)ethanol was identified by GC-MS, Figure S64)

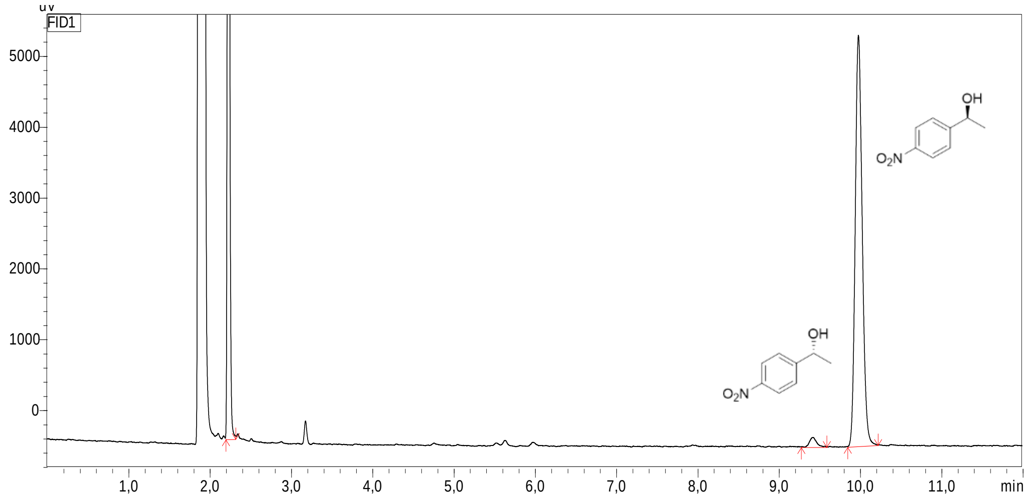

Figure S43. Chiral GC chromatogram of 4-ethylnitrobenzene reaction products with r*Aae*UPO and ADH-A, one-pot two-step cascade.

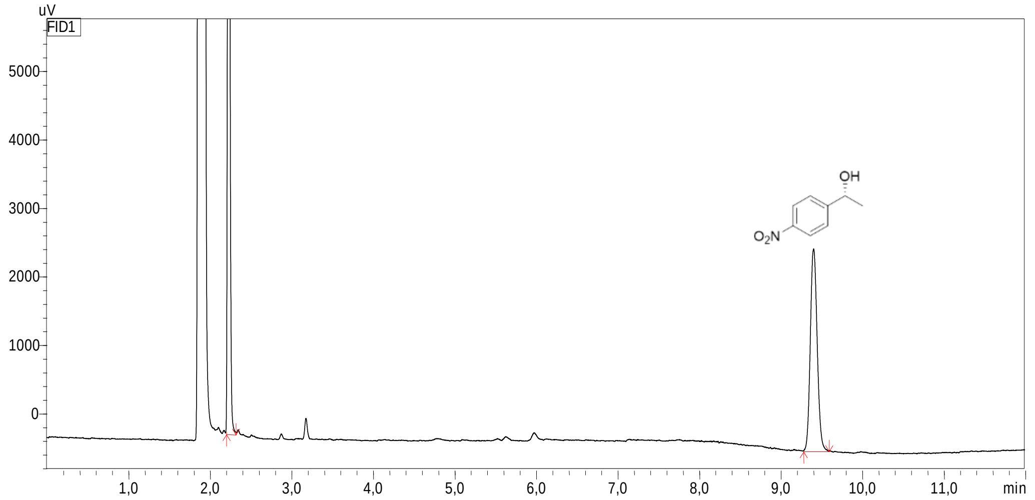

Figure S44. Chiral GC chromatogram of 4-ethylnitrobenzene reaction products with r*Aae*UPO and *Lk*ADH, one-pot two-step cascade.

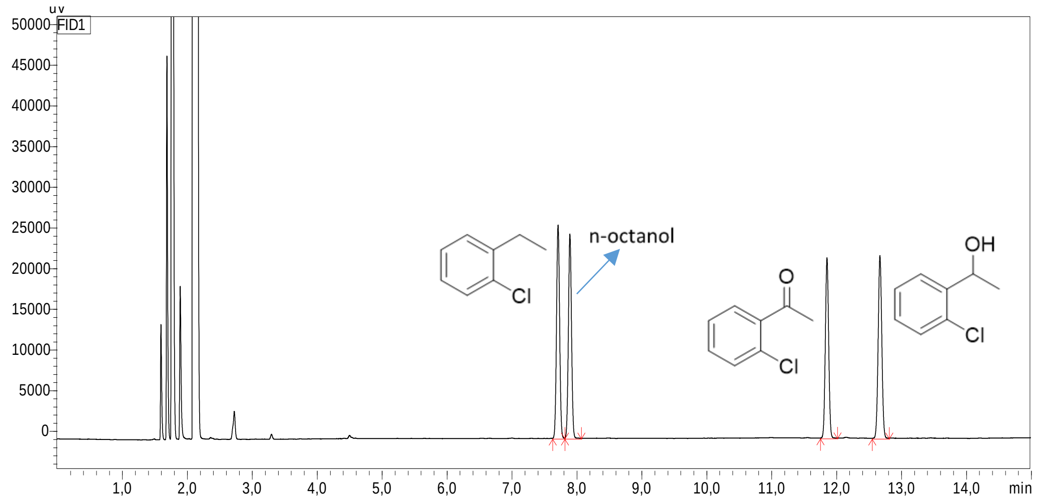

Figure S45. GC chromatogram of 1-chloro-2-ethylbenzene, 1-(2-chlorophenyl)ethanol, 2-chloroacetophenone commercial compounds.

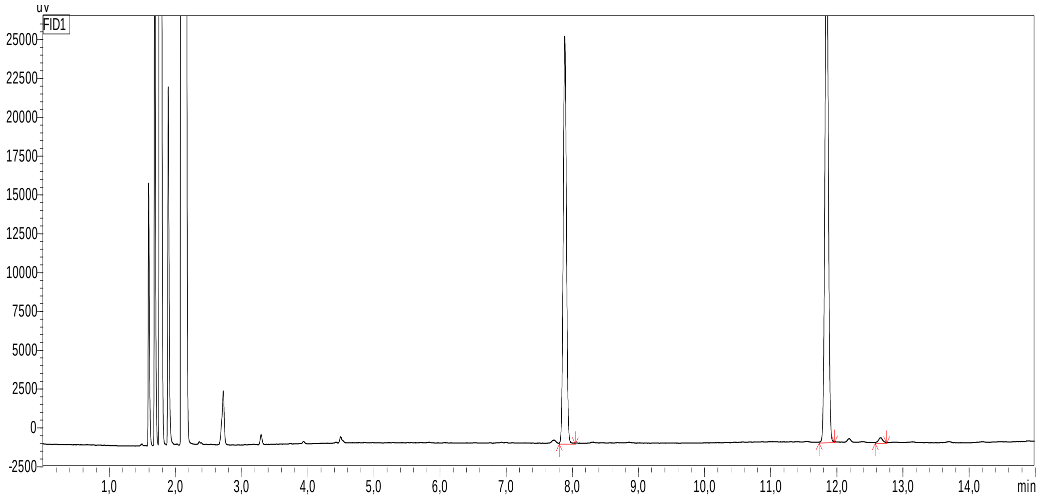

Figure S46. GC chromatogram of 1-chloro-2-ethylbenzene reaction products with r*Aae*UPO.

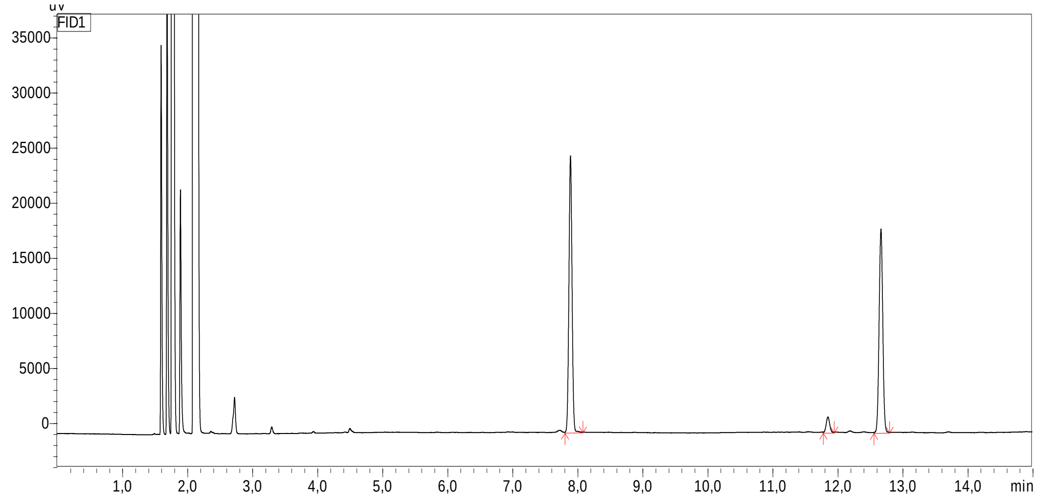

Figure S47. GC chromatogram of 1-chloro-2-ethylbenzene reaction products with r*Aae*UPO and ADH-A, one-pot two-step cascade.

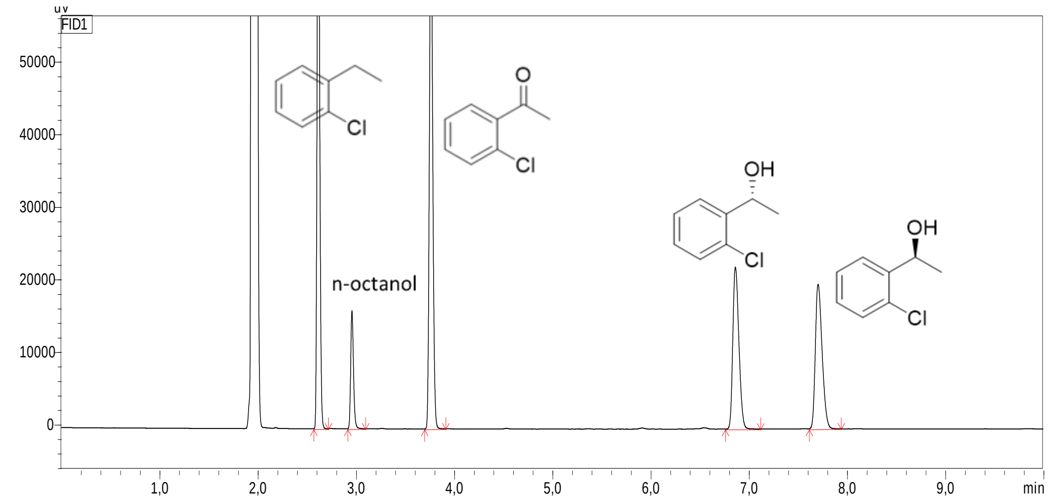

Figure S48. Chiral GC chromatogram of 1-chloro-2-ethylbenzene, 1-(2-chlorophenyl)ethanol, 2-chloroacetophenone commercial compounds.

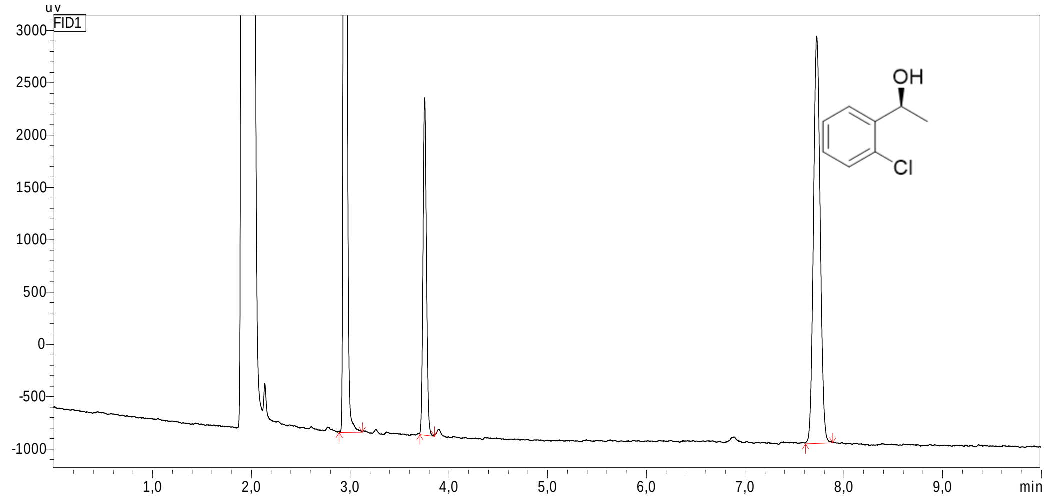

Figure S49. Chiral GC chromatogram of-1-chloro-2-ethylbenzene reaction products with r*Aae*UPO and ADH-A, one-pot two-step cascade.

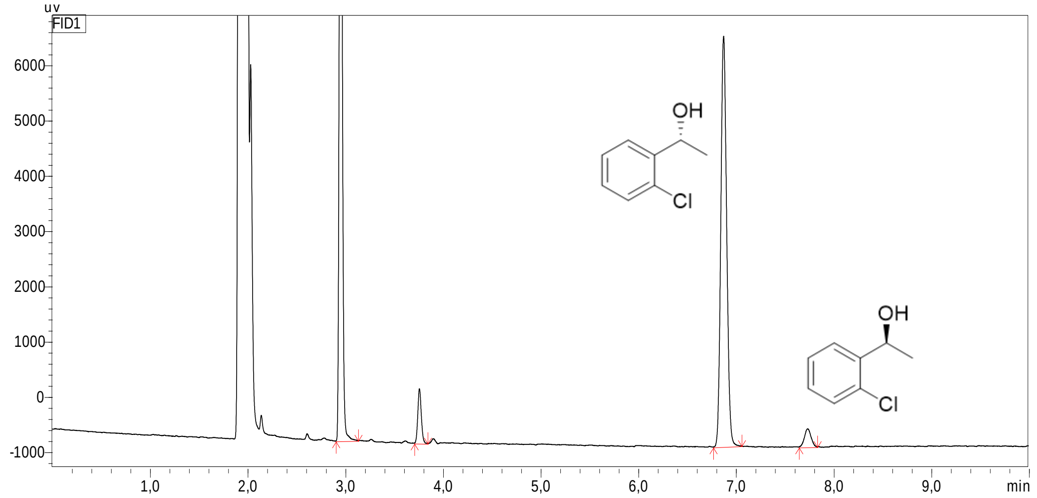

Figure S50. Chiral GC chromatogram of-1-chloro-2-ethylbenzene reaction products with r*Aae*UPO and *Lk*ADH, one-pot two-step cascade.

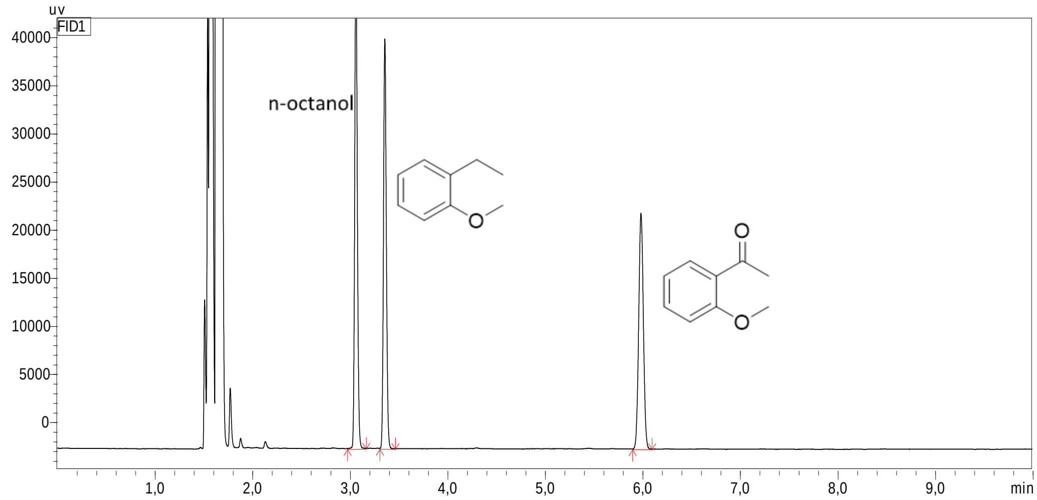

Figure S51. GC chromatogram of 2-ethylanisole, 2-methoxyacetophenone commercial compounds. (2-ethylanisole was identified by GC-MS, Figure S65)

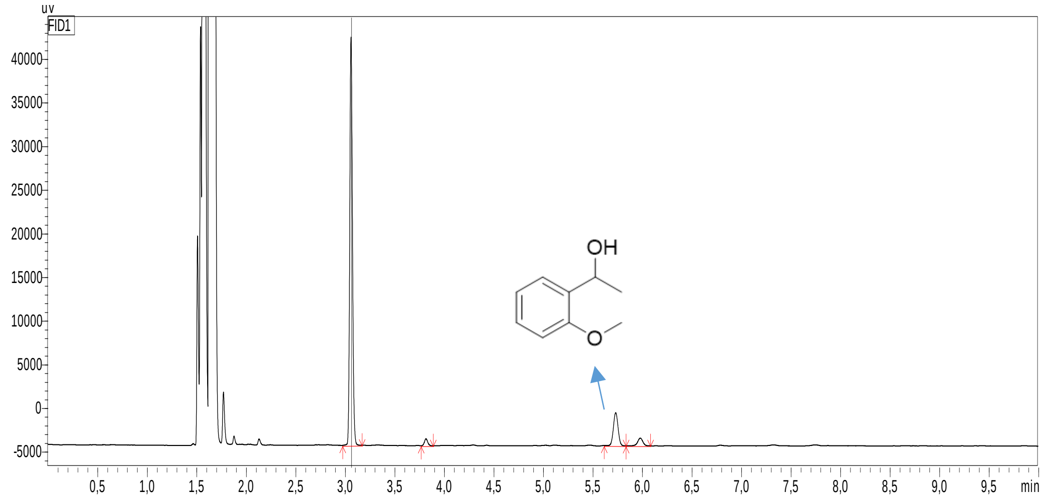

Figure S52. GC chromatogram of 2-ethylanisole reaction with r*Aae*UPO. (1-(2-methoxyphenyl)ethanol was identified by GC-MS, Figure S65)

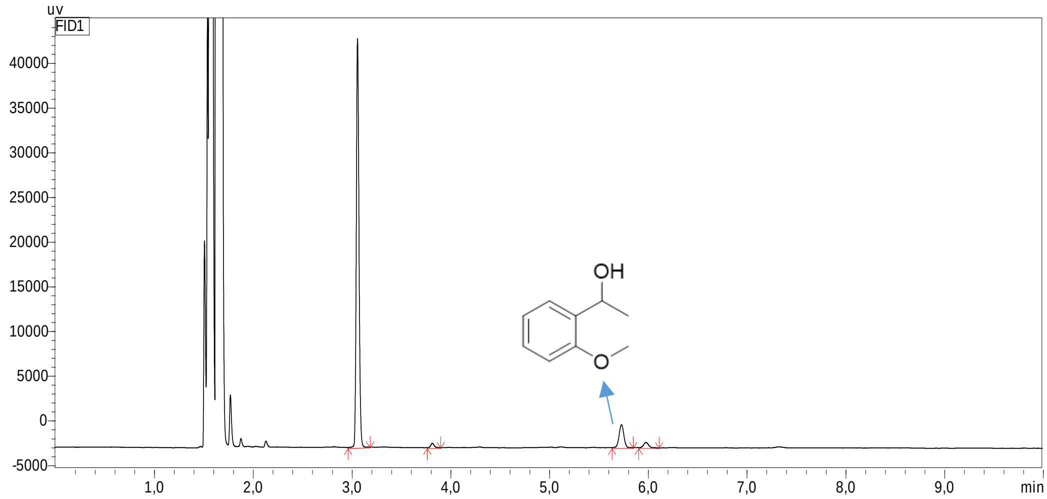

Figure S53. GC chromatogram of 2-ethylanisole reaction products with r*Aae*UPO and ADH-A one-pot two-step cascade.

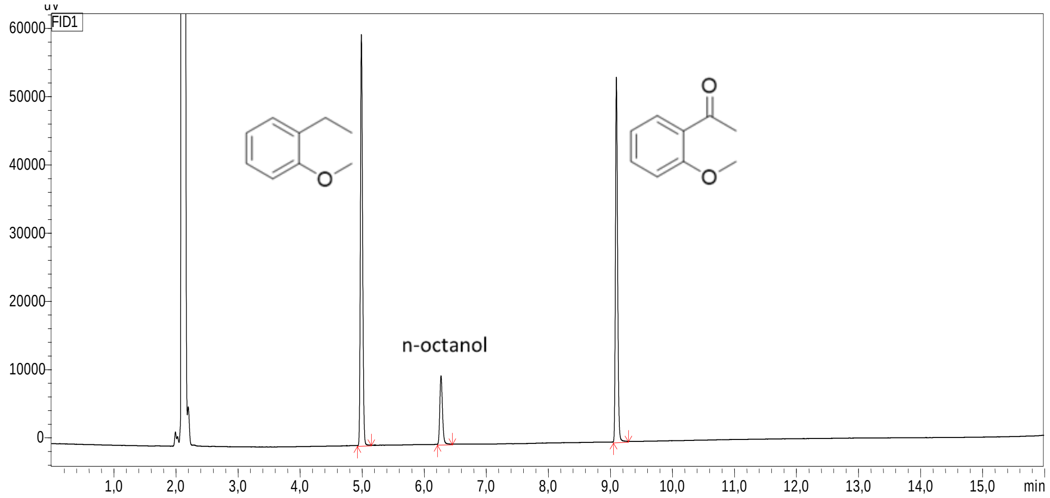

Figure S54. Chiral GC chromatogram 2-ethylanisole, 2-methoxyacetophenone commercial compounds. (2-ethylanisole was identified by GC-MS, Figure S65)

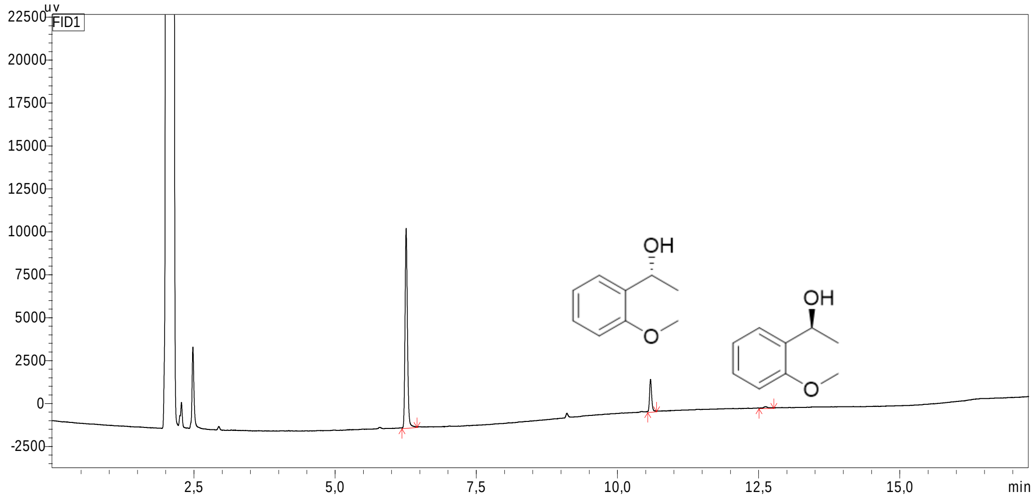

Figure S55. Chiral GC chromatogram 2-ethylanisole reaction products with r*Aae*UPO and ADH-A, one-pot two-step cascade.

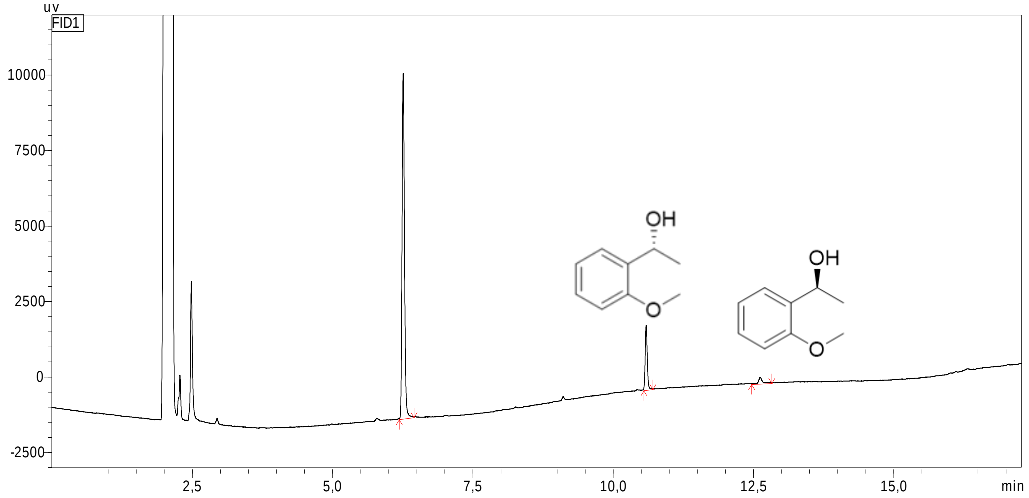

Figure S56. Chiral GC chromatogram of 2-ethylanisole reaction products with r*Aae*UPO and *Lk*ADH, one-pot two-step cascade.

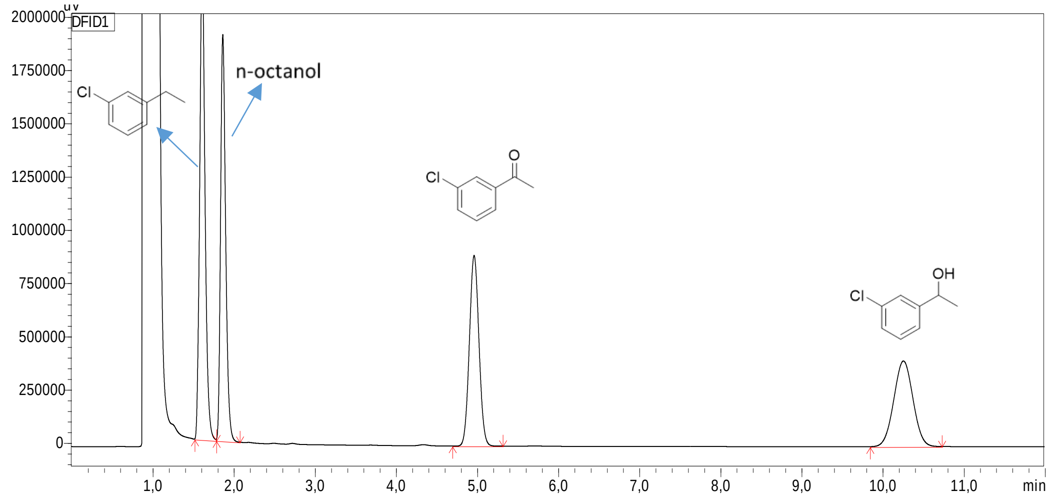

Figure S57. GC chromatogram of 1-chloro-3-ethylbenzene, 1-(3-chlorophenyl)ethanol, 3-chloroacetophenone commercial compounds.

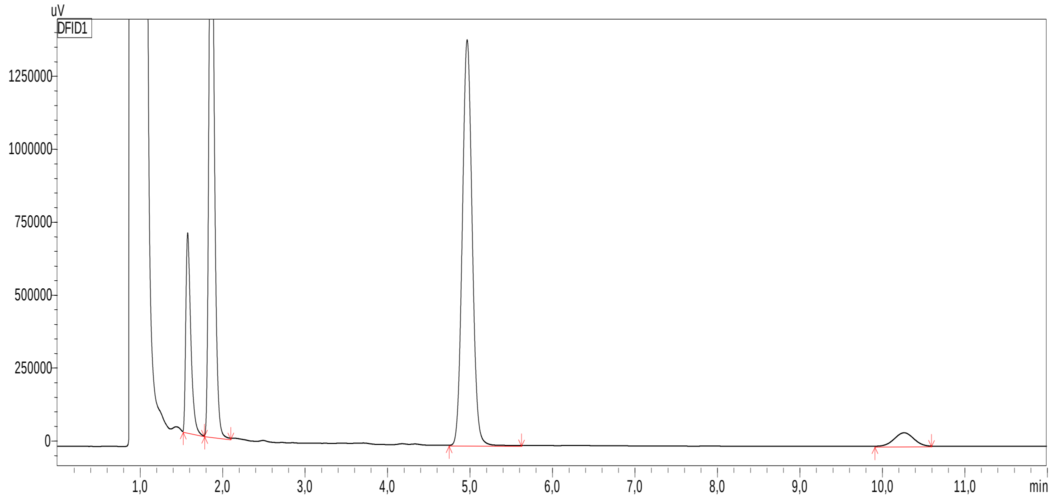

Figure S58. GC chromatogram of 1-chloro-3-ethylbenzene reaction products with r*Aae*UPO.

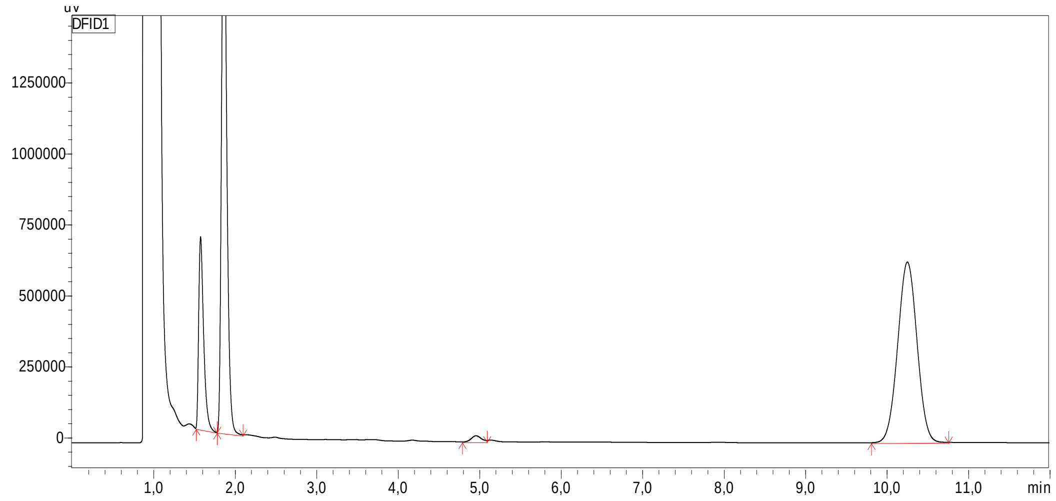

Figure S59. GC chromatogram of 1-chloro-3-ethylbenzene reaction products with r*Aae*UPO and ADH-A, one-pot two-step cascade.

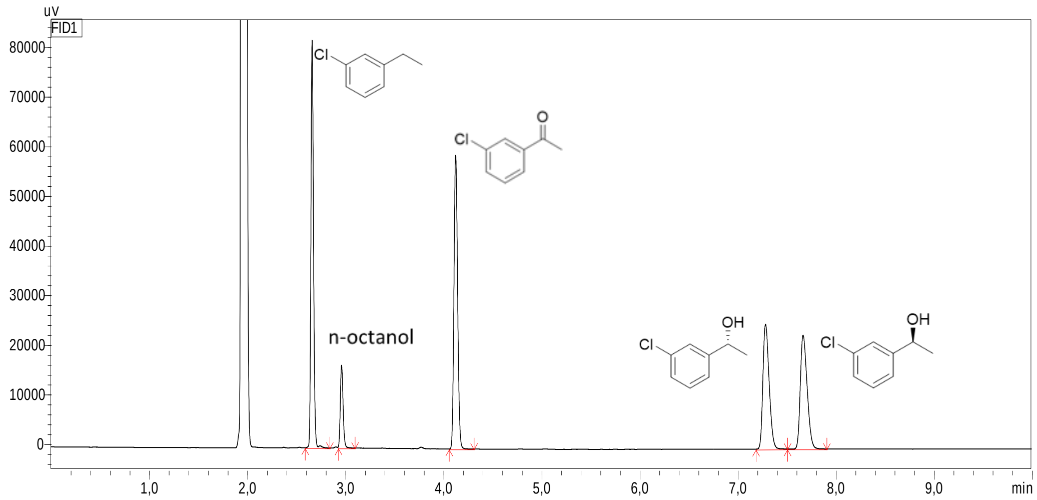

Figure S60. Chiral GC chromatogram of 1-chloro-3-ethylbenzene, 1-(3-chlorophenyl)ethanol, 3-chloroacetophenone commercial compounds.

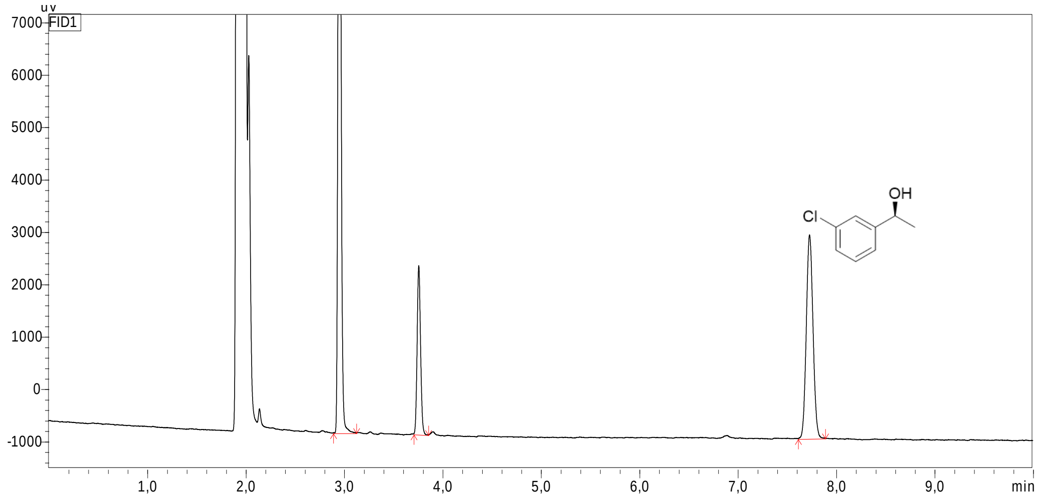

Figure S61. Chiral GC chromatogram of 1-chloro-3-ethylbenzene reaction products with r*Aae*UPO and ADH-A, one-pot two-step cascade.

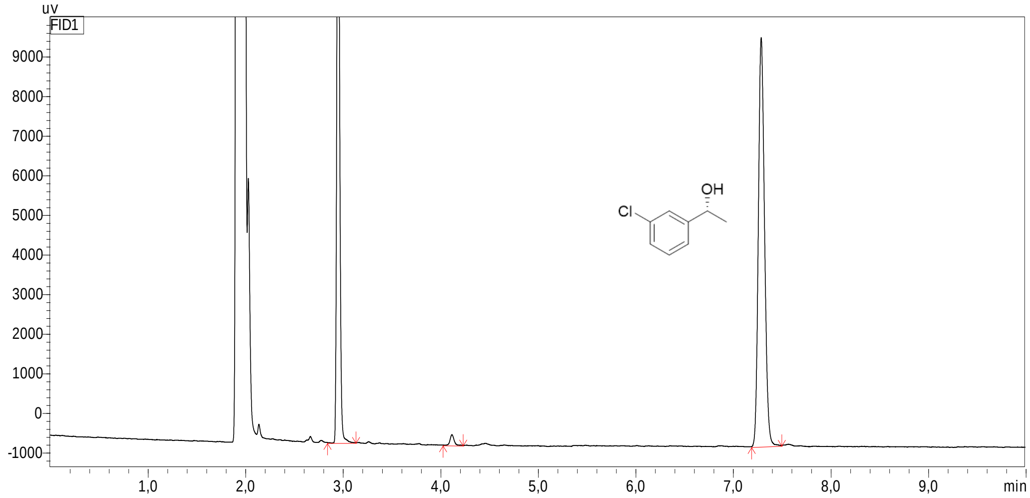

Figure S62. Chiral GC chromatogram of-1-chloro-3-ethylbenzene reaction products with r*Aae*UPO and *Lk*ADH, one-pot two-step cascade.

6. GC-MS chromatograms

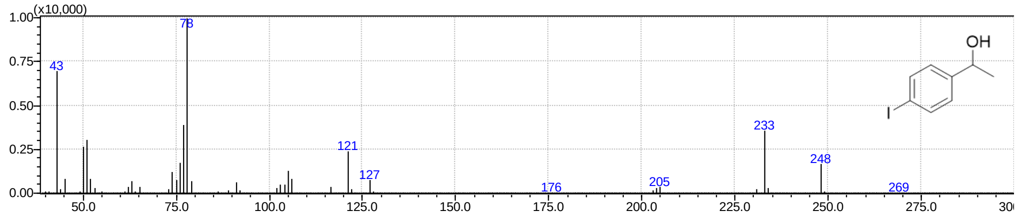

Figure S63. GC-MS mass spectrum 1-(4-iodophenyl)ethanol.

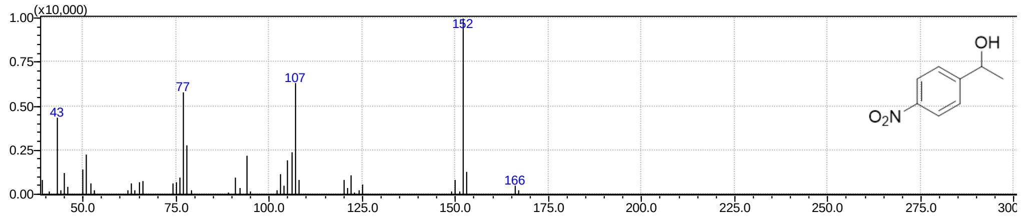

Figure S64. GC-MS mass spectrum 1-(4-nitrophenyl)ethanol.

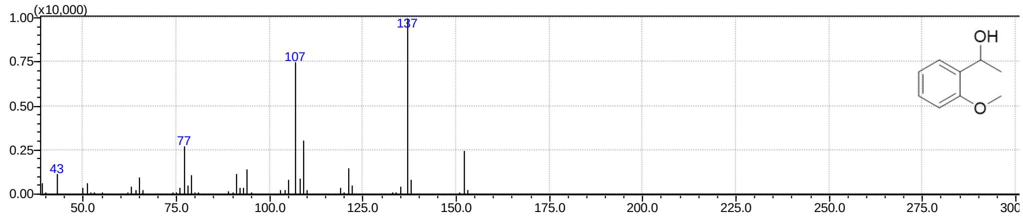

Figure S65. GC-MS mass spectrum 1-(2-methoxyphenyl)ethanol.

7. References

[1] a) P. Molina-Espeja, S. Ma, D. M. Mate, R. Ludwig, M. Alcalde, *Enzyme and Microbial Technology* **2015**, *73-74*, 29-33; b) P. Molina-Espeja, E. Garcia-Ruiz, D. Gonzalez-Perez, R. Ullrich, M. Hofrichter, M. Alcalde, D. Cullen, *Applied and Environmental Microbiology* **2014**, *80*(11), 3496-3507.

[2] a) K. Edegger, C. C. Gruber, T. M. Poessl, S. R. Wallner, I. Lavandera, K. Faber, F. Niehaus, J. Eck, R. Oehrlein, A. Hafner, W. Kroutil, *Chemical Communications* **2006**(22), 2402-2404; b) A. Weckbecker, W. Hummel, *Biocatalysis and Biotransformation* **2006**, *24*(5), 380-389.
